# Smartphone addiction, nomophobia, and neck-related functional disability among undergraduate students in a Nigerian University: a cross-sectional study

**DOI:** 10.1186/s12889-026-27976-z

**Published:** 2026-05-28

**Authors:** Jeneviv Nene John, Ujunwa Vivian Okonkwo, Sam Chidi Ibeneme, Gerhard Fortwengel, Blessing Chidimma Okpagu, Ezinne Olive Nwosu, Georgian Chiaka Ibeneme, Akachukwu Omumuagwula Nwosu, Nnenna Christiana Chinagozi-Amanze, Juliet Lucy Ekowa

**Affiliations:** 1 Department of Medical Rehabilitation, Faculty of Health Sciences & Technology, University of Nigeria, Enugu Campus, Enugu, Enugu State Nigeria; 2 Department of Physiotherapy, Faculty of Health Sciences, David Umahi Federal University of Health Sciences, Uburu, Ebonyi State Nigeria; 3 Department of Physiotherapy, Faculty of Health Sciences, School of Therapeutic Studies, University of the Witwatersrand, 7 York Road, Parktown, Johannesburg, Gauteng 2193 South Africa; 4 Faculty III, Hochschule Hannover University of Applied Sciences & Arts, Hannover, Germany; 5 Department of Medical Laboratory Science, Faculty of Health Sciences & Technology, University of Nigeria, Enugu Campus, Enugu, Enugu State Nigeria; 6 Department of Physiotherapy, University of Nigeria Teaching Hospital, Ituku- Ozalla, Enugu, Enugu state Nigeria; 7 Department of Community Health/Public Health Nursing, Faculty of Nursing Sciences, David Umahi Federal University of Health Sciences, Uburu, Ebonyi State Nigeria; 8 Praxis für ganzheitliche, Physiotherapie, Eichendorffstraße 5, Neckartailfingen, , Baden-Württemberg 72666 Germany

**Keywords:** Smartphone addiction, Nomophobia, Neck-related functional disability, Neck disability index, Text neck, Musculoskeletal disorders, University students, Digital health, Posture, Nigeria

## Abstract

**Background:**

The rapid increase in smartphone use among university students has raised concerns regarding digital dependence and associated musculoskeletal problems, particularly neck-related functional disability (NFD). However, the relative contributions of behavioural dependence (smartphone addiction and nomophobia) and ergonomic factors (posture and usage patterns) to neck disability remain insufficiently understood in low- and middle-income settings.

**Methods:**

A cross-sectional study was conducted among 399 undergraduate students at the University of Nigeria, Enugu Campus. Data were collected using a structured questionnaire and validated instruments: the Smartphone Addiction Scale–Short Version (SAS-SV), Nomophobia Questionnaire (NMP-Q), and Neck Disability Index (NDI). Descriptive statistics were computed for all variables. NDI scores were categorised to estimate prevalence of NFD. Pearson correlations were used to assess associations, with Spearman correlation conducted as a sensitivity analysis. Multiple linear regression models were fitted using log-transformed NDI (log NDI) as the outcome. Binary logistic regression was additionally performed to identify factors associated with moderate-to-severe disability (NDI ≥ 15). Statistical significance was set at *p* < 0.05.

**Results:**

Participants reported high smartphone use (6.18 ± 2.39 h/day). The mean smartpone addiction score was 35.60 ± 9.99, and the mean nomopobia score was 95.58 ± 31.57, indicating moderate-to-high digital dependence. The mean NFD score was 8.76 ± 6.38, with 82.7% of participants classified as having no or mild disability and 17.3% exhibiting moderate-to-severe disability. Smartphone addiction was significantly associated with NFD (*r* = 0.249, *p* < 0.001) and remained the strongest independent correlate in both linear (b = 0.0220, *p* < 0.001) and logistic regression models (OR = 1.04, 95% CI: 1.02–1.07). Neck flexion angle was also significantly associated with NFD. Nomophobia was correlated with smartphone addiction but was not an independent predictor of NFD.

**Conclusions:**

Smartphone addiction and forward-flexed neck posture were significantly associated with neck-related functional disability among university students. Nomophobia appears to reflect psychological dependence without direct physical impact. These findings highlight the need for integrated behavioural and ergonomic interventions to reduce digital-health risks in university settings.

**Supplementary Information:**

The online version contains supplementary material available at 10.1186/s12889-026-27976-z.

## Background

Smartphones have become integral to university life, reshaping how students communicate, learn, and access information [1, 2]. In higher education settings, students increasingly rely on smartphones for academic tasks, social interaction, and entertainment [3, 4]. This reliance is driven by the convenience and accessibility that smartphones offer, enabling students to engage with digital learning resources, participate in online lectures, and maintain social connections with peers and family. However, excessive and prolonged smartphone use, often accompanied by sustained forward-head posture, imposes abnormal mechanical load on the cervical spine and surrounding musculature [5, 6]. Such posture-related overuse has contributed to the emergence of “text neck,” a term commonly used to describe musculoskeletal symptoms associated with prolonged forward head posture during smartphone use, including neck pain, shoulder discomfort, upper back pain, muscle fatigue, stiffness, and, in some cases, headaches [6, 7].

A growing body of research links high volumes of smartphone use in young adults with musculoskeletal symptoms, particularly neck pain and disability [8, 9]. Among university students in Europe and the Middle East, high smartphone use and addiction (often ≥ 5 h/day) have been associated with neck pain, hand discomfort, and higher neck disability index (NDI) scores, indicating greater disruption of daily activities, work, and study [10, 11]. In such work, the NDI typically captures the functional impact of cervical symptoms, while posture and use variables such as duration of smartphone exposure, breaks during device use, and neck flexion angle characterize the “text neck” exposure context [12]. Studies in Italy and other European countries suggest that nomophobia, the fear or anxiety of being without one’s phone, is associated with unhealthy behaviors such as low physical activity and may mediate relationships between device dependence and adverse health outcomes [13, 14]. In Africa and Asia, similar patterns are emerging: in South Africa, more than half of undergraduate students report symptoms consistent with text neck, and smartphone addiction is positively correlated with functional impairment [15, 16]. in Nigeria, research among pre-service students shows that nomophobia and problematic phone use are common and closely related to addictive patterns [17]. Taken together, the literature points to a dual-pathway risk: one pathway driven by behavioural dependence (smartphone addiction and nomophobia) and another by physical strain (sustained forward head flexion and poor posture), both contributing to neck disability in university students.

Despite this growing global evidence, important gaps remain. Many studies examine either musculoskeletal outcomes (such as neck pain, posture, or NDI scores) or behavioural constructs, including smartphone addiction and nomophobia, in isolation, rather than integrating them within a combined predictive framework. In Nigeria and other African settings, research on smartphone addiction and nomophobia is increasing, and some studies have explored their associations with neck-related functional outcomes and posture-related risk factors among undergraduate populations [15, 17–19]; however, the overall literature remains limited. The prevalence of neck-related musculoskeletal complaints, the magnitude of NFD, and the extent to which behavioural dependencies predict these outcomes among Nigerian university students remain underexplored. Furthermore, few studies simultaneously consider smartphone addiction, nomophobia, and posture/use characteristics (such as neck flexion angle, duration of use, and break patterns) when modelling NFD, limiting understanding of how behavioural and ergonomic factors jointly contribute to neck-related musculoskeletal burden.

This study seeks to address these gaps by examining the prevalence and severity of NFD in the context of smartphone use and forward-flexed neck posture, the levels of smartphone addiction and nomophobia, and the predictive relationships between these behavioural constructs and NFD among university students in Nigeria. Specifically, the study objectives are to: (1) describe smartphone use patterns, posture-related behaviours, and NFD in a sample of undergraduate students; (2) quantify the associations between smartphone addiction, nomophobia, and NFD; and (3) evaluate the extent to which smartphone addiction and nomophobia predict NFD, over and above basic demographic and smartphone-use variables. By integrating behavioural dependence and posture-related risk within a single analytic framework, the findings are expected to inform public, digital and musculoskeletal health interventions that target both behavioural and ergonomic determinants of smartphone-related neck problems in university settings.

In this study, text neck is conceptualized as a posture-related exposure rather than a clinically diagnosed syndrome, referring to neck and upper-shoulder musculoskeletal symptoms arising in the context of habitual forward head posture during smartphone use. Neck-related functional disability, assessed using the NDI, is used as a proxy for functional limitation associated with smartphone-related neck strain, while smartphone use duration and neck flexion angle provide contextual indicators of exposure to “text neck” postures. As digital technologies increasingly shape academic routines, understanding these combined physical and behavioural risks has become a pressing public-health and digital-health priority [4, 20].

## Methods

### Study design

This study employed a cross-sectional survey design to examine how smartphone addiction and nomophobia are associated with NFD among undergraduate university students. The design allowed assessment of associations between behavioural dependence, posture-related exposures, and musculoskeletal outcomes at a single point in time, which is appropriate for digital health research in student populations. Reporting of the study follows the STROBE (Strengthening the Reporting of Observational Studies in Epidemiology) guidelines for cross-sectional studies.

### Study setting and population

The study was conducted at the University of Nigeria, Enugu Campus (UNEC), located in Enugu, southeastern Nigeria. UNEC is one of the two main campuses of the University of Nigeria and comprises seven faculties. The campus provides a suitable context for investigating digital health concerns because undergraduate students commonly use smartphones for academic activities (such as online lectures and access to digital learning resources), social interaction, and entertainment.

The target population comprised all full-time undergraduate students, aged 18 years and above, enrolled in any programme at the time of data collection. Students from all the faculties were eligible provided they reported regular smartphone use for academic or personal purposes. This population was chosen because they represent a digitally active group of young adults with high exposure to prolonged smartphone use and associated posture-related risks. Both male and female students across different academic years were included to capture variation in smartphone use habits and neck posture behaviours.

### Sampling strategy and sample size

A non-probability convenience sampling technique was used to recruit eligible students who were accessible and willing to participate during the data collection period. This approach was selected because the study was conducted within a large university setting, where students’ lecture schedules, clinical postings, and hostel availability varies considerably across faculties and academic levels. Convenience sampling is commonly used in exploratory, institution-based behavioural and digital-health surveys where the primary aim is to examine associations and generate context-specific evidence rather than produce nationally representative estimates [3, 8, 16, 18, 19]. Although this sampling method may limit external generalisability, it is widely regarded as pragmatic and appropriate for exploratory research in university populations. The required sample size was estimated using the Taro Yamane (1967) formula for finite populations [21]:

$$\:n=\frac{N}{1+N(e{)}^{2}}$$

where (N) is the population size and (e) is the margin of error. Based on official statistics from the Student Affairs Division, UNEC (2023), the total undergraduate population was 11,606 students. Assuming a 5% margin of error and a 95% confidence level, the minimum sample size was approximately 387 participants. To account for possible non-response and incomplete questionnaires, which is common in questionnaire-based surveys among university students, the calculated sample size was increased by 10% to ensure adequate final analyzable responses. Accordingly, the target sample size was increased to 426. In total, 399 students completed the survey, yielding a response rate of 93.7%.

**Fig. 1:**
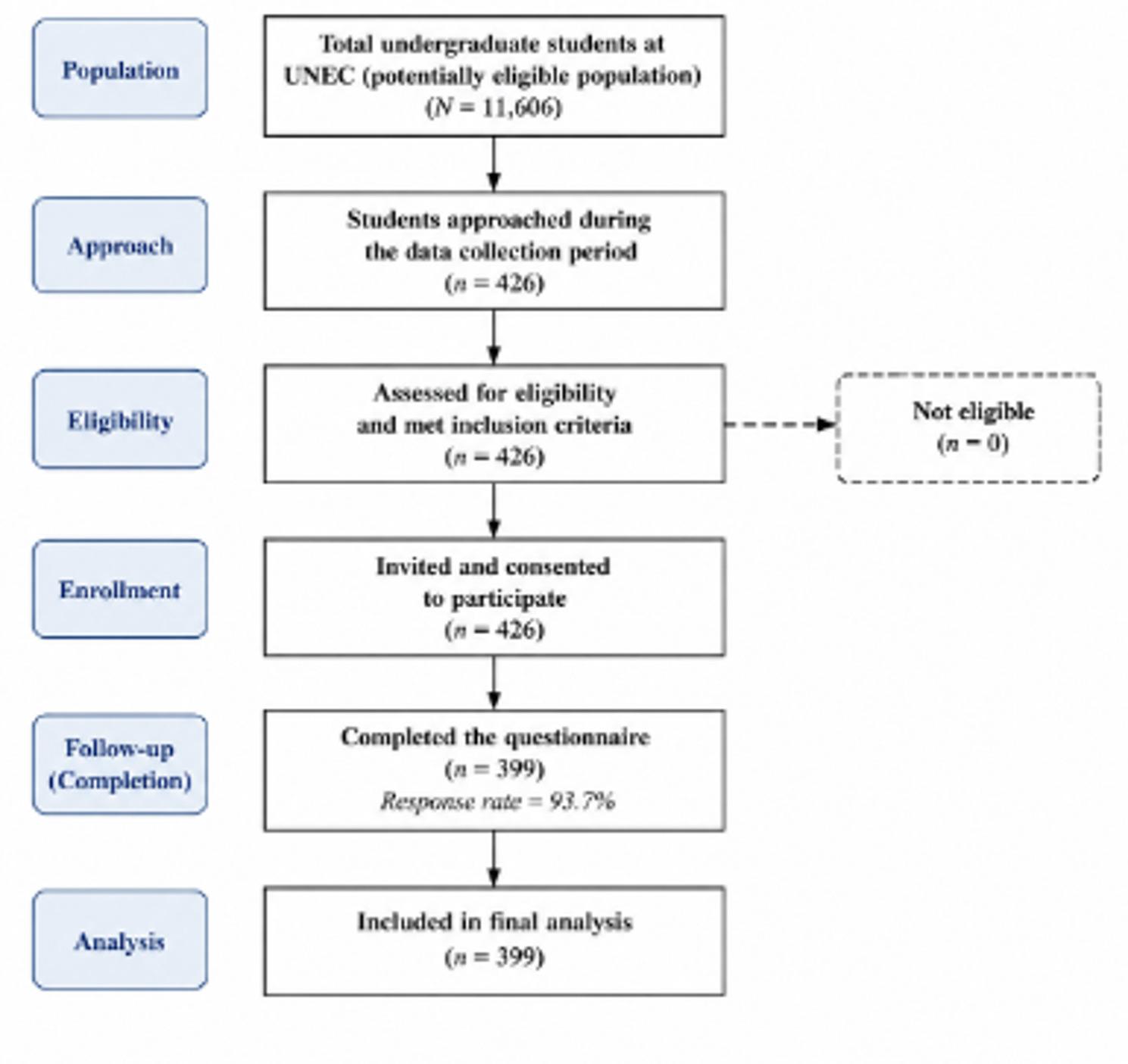
Flow diagram of participant recruitment and inclusion in the study

To provide a clear overview of participant progression through the study, the following numbers were recorded at each stage of recruitment: a total of 11,606 undergraduate students constituted the potentially eligible population; 426 students were approached during the data collection period; all 426 individuals met the inclusion criteria and were invited to participate; all invited students consented and were included in the study; 399 students successfully completed the questionnaire; and all 399 completed responses were included in the final analysis. A flow diagram illustrating the stages of participant recruitment and eligibility assessment is presented in Fig. 1.

### Eligibility criteria

Inclusion criteria were: full-time undergraduate students aged ≥ 18 years, regular smartphone use for at least one hour per day (for academic or personal purposes), and willingness to provide informed consent and complete the questionnaire. Students were excluded if they were postgraduate students; reported diagnosed cervical spine pathology, significant cervical trauma, or congenital cervical disorders; had systemic neurological or musculoskeletal conditions known to cause neck pain (e.g. cervical disc disease, spinal cord injury); were currently receiving treatment for neck pain or disability; or reported severe psychological or neurological conditions that could interfere with reliable questionnaire completion. Importantly, this study did not diagnose text neck syndrome clinically or using symptom-based criteria; instead, it examined NFD and associated behavioural and posture-related factors.

### Instruments and measures

The questionnaire used in this study comprised two sections: (1) investigator-developed items assessing socio-demographic characteristics and smartphone-use behaviours; and (2) three previously published and validated instruments, namely the Smartphone Addiction Scale–Short Version (SAS-SV) [25], the Nomophobia Questionnaire (NMP-Q) [31], and the Neck Disability Index (NDI) [34].

#### Socio-demographic and smartphone-use patterns

The first section of the questionnaire was developed by the investigators to capture sociodemographic characteristics and smartphone-use behaviours relevant to the study objectives. Items assessed age, sex, faculty, department/programme, academic level, average daily smartphone-use duration, break frequency during smartphone use, and usual neck posture during smartphone use. Neck posture was assessed using a visual selection item with five posture categories: 0° (neutral), 15° (slight tilt), 30° (mild bend), 45° (moderate bend), and 60° (pronounced bend). This visual posture item was designed to provide a pragmatic estimate of habitual neck flexion during smartphone use in a field-survey context, rather than a biomechanical diagnosis. Grounded in previous ergonomics and posture research, this visual-angle approach offers a semi-quantitative estimate of neck flexion in population-based studies and provides contextual insight into “text neck” exposure [22–24].

The Investigator-developed items were reviewed by subject experts for clarity, face validity, and relevance to the study objectives. The questionnaire was then pilot-tested among 30 undergraduate students who were not included in the final analysis. The pilot study assessed item clarity, ease of comprehension, appropriateness of response options, and completion time. Minor wording and layout refinements were made following the pilot feedback. Because the investigator-developed items assessed heterogeneous behavioural and descriptive constructs rather than a single latent psychological scale, cronbach’s alpha was not calculated for this section. Internal consistency was instead reported for the other validated psychometric instruments used in the study, including the SAS-SV, NMP-Q and NDI. The absence of formal psychometric validation of the investigator-developed smartphone-use items has been acknowledged as a limitation. An English language version of the investigator-developed questionnaire is provided as a supplementary file.

#### Smartphone addiction

Smartphone addiction severity was assessed using the SAS-SV, a 10-item self-report instrument [25]. Participants were asked to indicate the extent to which each statement applied to them (e.g. “I miss planned work due to smartphone use”), using a 6-point Likert scale ranging from 1 (strongly disagree) to 6 (strongly agree). Total scores range from 10 to 60, with higher scores indicating greater risk of smartphone addiction [25]. Consistent with previous studies, gender-specific cut-offs (≥ 31 for males and ≥ 33 for females) were used to classify participants at risk of smartphone addiction [26, 27]. In the present sample, internal consistency of the SAS-SV was high (Cronbach’s α = 0.883 overall; 0.895 for males; 0.872 for females). Prior research has demonstrated high internal consistency (α ≈ 0.81–0.91), strong construct validity, and satisfactory structural reliability in bifactor analyses [28, 29], and the scale has been successfully adapted for Nigerian university students, supporting its use in this context [18, 30].

#### Nomophobia

Nomophobia was measured using the NMP-Q, a 20-item self-report instrument assessing fear or anxiety associated with being unable to use one’s mobile phone [31]. Items are rated on a 7-point Likert scale from 1 (strongly disagree) to 7 (strongly agree), yielding total scores where higher values indicate greater nomophobic tendencies. Previous validation studies in student populations report excellent internal consistency (Cronbach’s α ≈ 0.94–0.96), strong construct validity, and a stable four-factor structure, supporting its use as a measure of psychological dependence on mobile devices [32, 33].

#### Neck-related functional disability

Neck-related functional disability, representing functional limitation associated with smartphone-related neck strain, was assessed using the NDI. The NDI is a widely used 10-item instrument measuring disability in daily activities such as pain intensity, personal care, lifting, reading, driving, sleeping, work, concentration, recreation, and headaches [34]. Each item is scored from 0 to 5, yielding a total score of 0–50. Scores are interpreted as follows: 0–4 (no disability), 5–14 (mild disability), 15–24 (moderate disability), 25–34 (severe disability), and ≥ 35 (complete disability), representing increasing levels of functional limitation. The NDI has shown strong internal consistency, high test–retest reliability (intraclass correlation coefficient ≈ 0.88–0.97), and good construct validity across populations, including Nigerian cohorts [35, 36], making it appropriate for assessing functional impacts associated with posture and smartphone use.

### Data collection procedure

Ethical approval was obtained from the Health Research Ethics Committee (Certificate No. NHREC/05/01/2008B–FWA00002458–IRB00002323). The study adhered to the principles of the Declaration of Helsinki, and all participants provided written informed consent. Participation was voluntary, and confidentiality was strictly maintained. Data were collected over a four-month period during the academic term. Trained research assistants visited lecture halls, the university library, and student hostels to recruit participants. The aims and procedures of the study were explained, and eligible students were invited to participate. Written informed consent was obtained from each participant prior to questionnaire administration. Students completed the paper-based questionnaire on-site in a single session. Immediately after completion, research assistants checked each questionnaire for missing or unclear responses and clarified any issues with the participant to minimise data loss and enhance reliability. Completed questionnaires were assigned unique identification numbers, anonymised, and stored securely in locked cabinets and encrypted electronic files.

### Statistical analysis

All statistical analyses were conducted using IBM SPSS Statistics version 25.0. Descriptive statistics were first generated to summarise the sample characteristics and key study variables. Continuous measures including age, smartphone addiction, nomophobia, NFD, and average daily smartphone use duration were examined using means, standard deviations, medians, and ranges. Categorical variables such as sex, academic level, and break frequency during smartphone use were summarised as counts and percentages. Given the wide variability in reported average daily smartphone use duration, the variable was categorised into four exposure groups (low, moderate, high, and very high use) as shown in Table 1, to allow for clinically interpretable subgroup analysis and to account for potential non-linear effects. This categorisation was used for subgroup and supplementary regression analyses to explore potential non-linear exposure effects.

**Table 1:** Categorisation of daily smartphone use duration

Category	Hours/day	Interpretation
Low use	1–3	Minimal exposure
Moderate use	4–6	Typical use
High use	7–9	Elevated exposure
Very high use	≥ 10	Excessive exposure

**Fig. 2:**
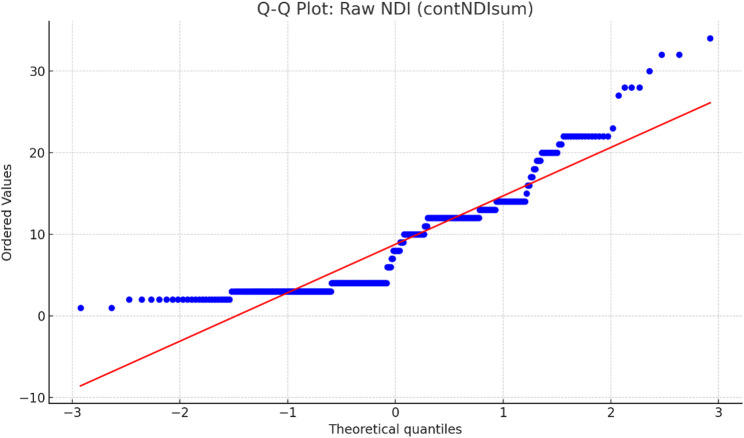
Q-Q plot of raw NFD scores showing deviation from normality

**Fig. 3:**
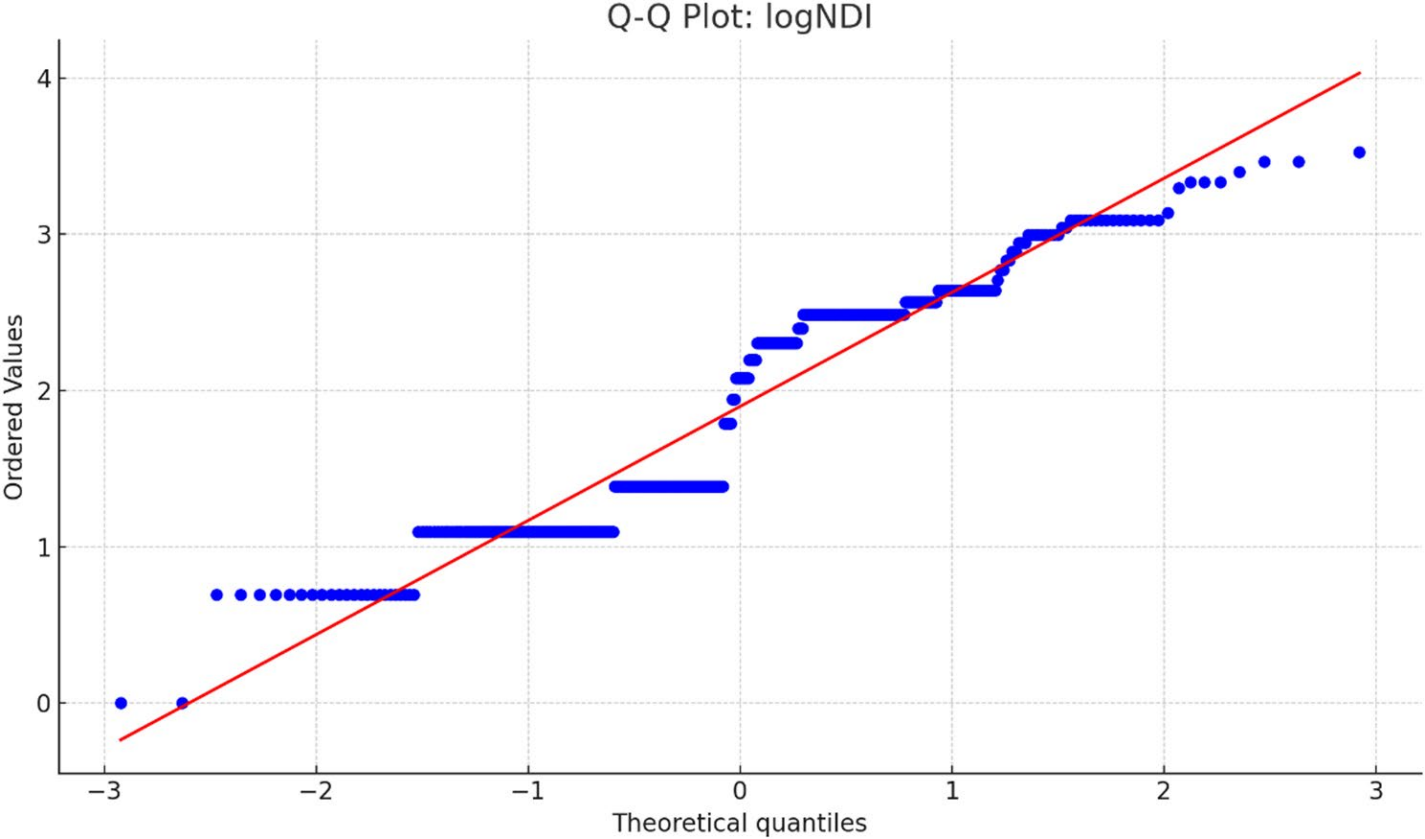
Q-Q plot of log-transformed NFD (log NFD) scores demonstrating improved approximation to normal distribution

Before conducting inferential analyses, the distributional properties of continuous variables were assessed to ensure that the assumptions for parametric procedures were met. Normality was evaluated using the shapiro-wilk test, supported by inspection of skewness and kurtosis indices, histogram plots, and Q–Q plots. The NFD variable demonstrated significant positive skew (Fig. 2); therefore, a logarithmic transformation was applied to generate log NFD (Fig. 3), which exhibited substantially improved symmetry and was subsequently used as the dependent variable in all regression models. Smartphone addiction and nomophobia scores showed only mild deviations from normality and were retained in their original forms.

Pearson correlation coefficients were used as the primary measure of linear association among continuous variables. Given the skewed distribution of NFD and the ordinal nature of some behavioural variables, Spearman’s rank correlation coefficients were additionally calculated as a sensitivity analysis to assess the robustness of the associations. Correlation strength was interpreted using Cohen’s conventional thresholds: small (r/ρ ≈ 0.10), moderate (r/ρ ≈ 0.30), and large (r/ρ ≥ 0.50). Statistical significance was set at *p* < 0.05 (two-tailed). These analyses addressed Objective 2 of the study.

To evaluate the multivariable associations between smartphone addiction, nomophobia, and NFD (Objective 3), multiple linear regression models were fitted using log NFD as the outcome variable. An initial full model included smartphone addiction, nomophobia, average daily smartphone use duration, break frequency, neck flexion angle, sex, age, and academic level. To identify the most parsimonious and best-fitting predictors, stepwise procedures were employed, including forward selection, backward elimination, and bidirectional stepwise selection. Competing models were compared using the coefficient of determination (R²), adjusted R², Akaike Information Criterion (AIC), and Bayesian Information Criterion (BIC). Model diagnostics were performed to verify adherence to regression assumptions, including assessment of residual normality using Q–Q plots and the shapiro–wilk test, evaluation of homoscedasticity via residuals-versus-fitted plots, examination of multicollinearity using variance inflation factors, and checks for influential observations. A significance level of α = 0.05 was applied throughout.

To provide clinically interpretable estimates of risk, NFD scores were dichotomised into no/mild disability (NFD < 15) and moderate-to-severe disability (NFD ≥ 15), and binary logistic regression was subsequently performed to identify predictors of clinically meaningful neck disability. Smartphone use duration was also analysed as a categorical variable in supplementary regression models to explore potential non-linear associations with NFD.

## Results

### Sample characteristics

A total of 399 undergraduate students participated in the study. The mean age was 24.0 ± 1.37 years (range: 19–29), and 53.8% were males. Participants were drawn from all faculties and academic levels (years 1–5) of the University of Nigeria, Enugu Campus. The academic level of participants showed broad representation across programme stages, with the highest proportion in the fifth year (37.3%) and the lowest in the first year (7.5%). Demographic characteristics are presented in Table 2.

**Table 2:** Demographic characteristics of participants (*N* = 399)

Variable	Mean (SD)	*N* (%)	Median	Range (Min–Max)
Age (years)	24.02 (1.37)		24	19–29
Gender				
female		184 (46.2)		
male		215 (53.8)		
Academic level				
Fifth year		149 (37.3)		
Fourth year		90(22.6)		
Third year		73(18.3)		
Second year		57(14.3)		
First year		30(7.5)		
Faculty				
Basic Medical Sciences		44(11.0)		
Business Administration		42(10.5)		
Health Sciences and Technology		175(43.9		
Dentistry		15(3.8)		
Environmental		31(7.8)		
Law		51(12.8)		
Medical sciences		41(10.3)		

### Objective 1: descriptive analysis of smartphone use, posture behaviours, and neck-related functional disability

Data from 399 participants were included in the analysis. Undergraduate students demonstrated high smartphone use, moderate to high smartphone addiction, elevated nomophobia, and predominantly mild but increasingly significant NFD (Tables 3, 4, 5 and 6). Forward-flexed neck posture was common and worsened in higher academic years. Faculty and academic level analyses confirmed consistent escalation in digital dependence and musculoskeletal symptoms, particularly in years 4 and 5. The mean NFD score was 8.76 (SD 6.38; range 1–34), indicating generally low to moderate NFD in the sample. The mean smartphone addiction score was 35.60 (SD 9.99; range 10–58), and the mean nomophobia score was 95.58 (SD 31.57; range 17–139) (Table 3).

**Table 3:** Descriptive statistics for key continuous variables (*N* = 399)

Variable	Mean (SD)	Median	Range (Min –Max)	P25	P75
Hours of smartphone use/day	6.18 (2.39)	6	1–12	–	–
Smartphone addiction	35.60 (9.99)	36	10–58	29.0	43.0
Nomophobia	95.58 (31.57)	96	17–139	73.0	126.0
Neck-related functional disability	8.76 (6.38)	8	1–34	3.0	12.0

Values are presented as mean (standard deviation), median, and range (minimum–maximum). Where available, the 25th (P25) and 75th (P75) percentiles are also reported to describe data dispersion

**Table 4:** Smartphone use patterns by faculty (hours/day)

Faculty	Hours/Day		*n*
	Mean	SD	
Dentistry	7.87	2.53	15
Business administration	6.40	2.40	42
Health Sciences and Technology	6.19	2.58	175
Environmental studies	6.52	2.10	31
Medical Sciences	6.37	1.97	41
Law	5.96	2.61	51
Basic Medical Sciences	5.95	2.12	44

Values represent mean daily smartphone use (hours/day) with standard deviations. Mean daily smartphone use by faculty. Students in Dentistry, Medical Sciences, and Business Administration recorded the highest daily usage, whereas those in Basic Medical Sciences and Law reported the lowest average use

**Table 5:** Smartphone use by academic level

Academic Level	Hours/Day		*n*
	Mean	SD	
Year 1	6.07	1.74	30
Year 2	6.28	2.49	57
Year 3	6.15	2.36	73
Year 4	6.41	2.42	90
Year 5	6.13	2.47	149

**Table 6:** Smartphone use patterns, posture-related behaviours, and neck-related functional disability among undergraduate students (*N* = 399)

Variable	Measure	Mean (SD)	Range/ Distribution	Notes
Smartphone Use Patterns	Daily smartphone use (hours/day)	6.18 (2.39)	1–12 h	High across all years; peaks in Years 4–5
	Break frequency	–	Modal response = “Sometimes”	Indicates inconsistent ergonomic breaks
Posture-Related Behaviours	Neck flexion angle	–	30° most common; followed by 45° and 60°	Majority exhibit forward-flexed posture
NFD	NFD total score	8.76 (6.38)	1–34	Mild disability overall; moderate disability in subset
Smartphone addiction	Smartphone Addiction	35.60 (9.99)	10–58	Moderate–high addiction levels
	Nomophobia	95.58 (31.57)	17–139	Moderate–severe nomophobia in most students
Demographics	Age (years)	24.02 (1.37)	19–29	Balanced age range
	Academic level	3.68 (1.31)	1–5	Majority in years 3–5
	Sex	–	53.8% male, 46.2% female	Balanced representation

*NFD* Neck-related Functional Disability

#### Smartphone use patterns

Daily smartphone use was high across the sample, with students reporting an average of 6.18 ± 2.39 h per day (range: 1–12 h) (Table 6). To account for the wide variability in average daily smartphone use, usage duration was categorised into four exposure groups. As shown in Table 7, the majority of participants fell within the moderate (44.1%) and high-use (33.6%) categories, indicating that most students engaged in prolonged daily smartphone use. A smaller proportion reported very high use (11.8%), representing individuals at potentially elevated behavioural and ergonomic risk, while 10.5% were classified as low-use users. This categorisation highlights substantial heterogeneity in smartphone exposure within the sample and provides a clinically interpretable framework for assessing risk.

**Table 7:** Distribution of daily smartphone use categories (*N* = 399)

Category	Hours/day	Interpretation
Low use	1–3	42 (10.5%)
Moderate use	4–6	176 (44.1%)
High use	7–9	134 (33.6%)
Very high use	≥ 10	47 (11.8%)
Total	-	399 (100%)

Faculty-level differences were observed in smartphone use patterns, with the highest daily use reported in Faculties of Dentistry, Medical Sciences, Business Administration and Health Sciences and Technology (Table 4). Usage increased progressively from Year 1 to Year 4, peaking in Year 4, and remained high in Year 5 (Table 5).

Break-taking behaviour during smartphone use varied widely; however, the modal response was “sometimes,” indicating inconsistent ergonomic break habits. Smartphone-use characteristics are presented in Table 6.

Most students reported using their smartphones in forward-flexed neck postures (Table 6). The 30° flexion angle was the most frequently selected, followed by 45° and 60°, indicating widespread adoption of ergonomically risky positions. Neutral (0°) posture was rarely reported. Posture severity increased with academic level, particularly in years 4 and 5, suggesting cumulative ergonomic exposure as academic demands and digital engagement intensified.

#### Digital dependence: smartphone addiction and nomophobia

Smartphone addiction scores indicated moderate to high behavioural dependence, with a mean score of 35.60 ± 9.99. Higher addiction levels were observed in:

Business Administration, Dentistry and Medical Sciences (Table 8). Nomophobia scores were also elevated (mean: 95.58 ± 31.57), reflecting moderate to severe anxiety related to being without one’s smartphone. Nomophobia increased steadily from year 1 to a peak in year 4 (Table 9).

**Table 8:** Smartphone addiction, nomophobia, and neck-related functional disability by faculty (*N* = 399)

Faculty	Smartphone addiction Mean (SD)	Nomophobia Mean (SD)	NFD Mean (SD)	*n*
Basic Medical Sciences	31.30 (11.77)	86.52 (36.96)	8.48 (5.72)	44
Business Administration	36.14 (9.40)	102.29 (25.76)	9.26 (5.96)	42
Dentistry	35.00 (11.44)	88.67 (27.65)	7.47 (4.45)	15
Environmental Studies	34.77 (9.41)	93.90 (29.57)	9.48 (6.19)	31
Health Sciences and Technology	33.37 (9.36)	96.74 (32.67)	9.08 (6.96)	175
Law	35.78 (9.60)	94.41 (29.87)	9.04 (5.79)	51
Medical Sciences	35.30 (8.14)	84.15 (27.13)	7.95 (5.83)	41

*NFD* Neck-related Functional Disability

**Table 9:** Smartphone addiction, nomophobia, neck-related functional disability, and hours of use by academic level

Academic Level	Smartphone addiction Mean (SD)	Nomophobia Mean (SD)	NFD Mean (SD)	Hours/day Mean (SD)	*n*
Year 1	33.97 (13.98)	85.83 (39.48)	8.40 (5.15)	6.07 (1.74)	30
Year 2	33.67 (10.02)	95.74 (29.67)	8.28 (6.20)	6.28 (2.49)	57
Year 3	34.84 (9.46)	97.84 (29.47)	8.07 (6.19)	6.15 (2.36)	73
Year 4	37.11 (9.17)	101.61 (31.12)	9.36 (6.91)	6.41 (2.42)	90
Year 5	36.13 (9.71)	92.74 (31.38)	9.15 (5.82)	6.13 (2.47)	149

*NFD* Neck-related functional Disability

#### Neck-related functional disability

The mean NFD score was 8.76 ± 6.38, indicating predominantly mild NFD in the overall sample. However, a subset of participants scored within the moderate disability range. To provide clinically interpretable estimates of neck-related functional impairment, NFD scores were further categorised using standard clinical thresholds (Table 10). The majority of participants fell within the no to mild disability categories (82.7%), indicating generally low levels of functional limitation. Specifically, 33.1% of students reported no disability, while 49.6% demonstrated mild disability. A smaller proportion exhibited moderate disability (13.5%) and severe disability (3.8%), with no cases of complete disability observed. These findings suggest that although overall neck disability was predominantly mild, a meaningful subset of students experienced clinically relevant functional limitations.

**Table 10:** Prevalence of NFD (NFD categories)

NFD Category	Score Range	*n* (%)
No disability	0–4	132 (33.1%)
Mild disability	5–14	198 (49.6%)
Moderate disability	15–24	54 (13.5%)
Severe disability	25–34	15 (3.8%)
Complete disability	≥ 35	0 (0.0%)
Total	—	399 (100%)

Higher disability levels were found among students in Faculties of Dentistry, Health Sciences and Technology, Law, Environmental Studies and Business Administration (Table 8). Progressively higher NFD scores were observed from Years 1 to 5, with Years 4 and 5 showing the greatest neck disability (Table 9).

#### Faculty-level and academic-level integrated patterns

**Fig. 4:**
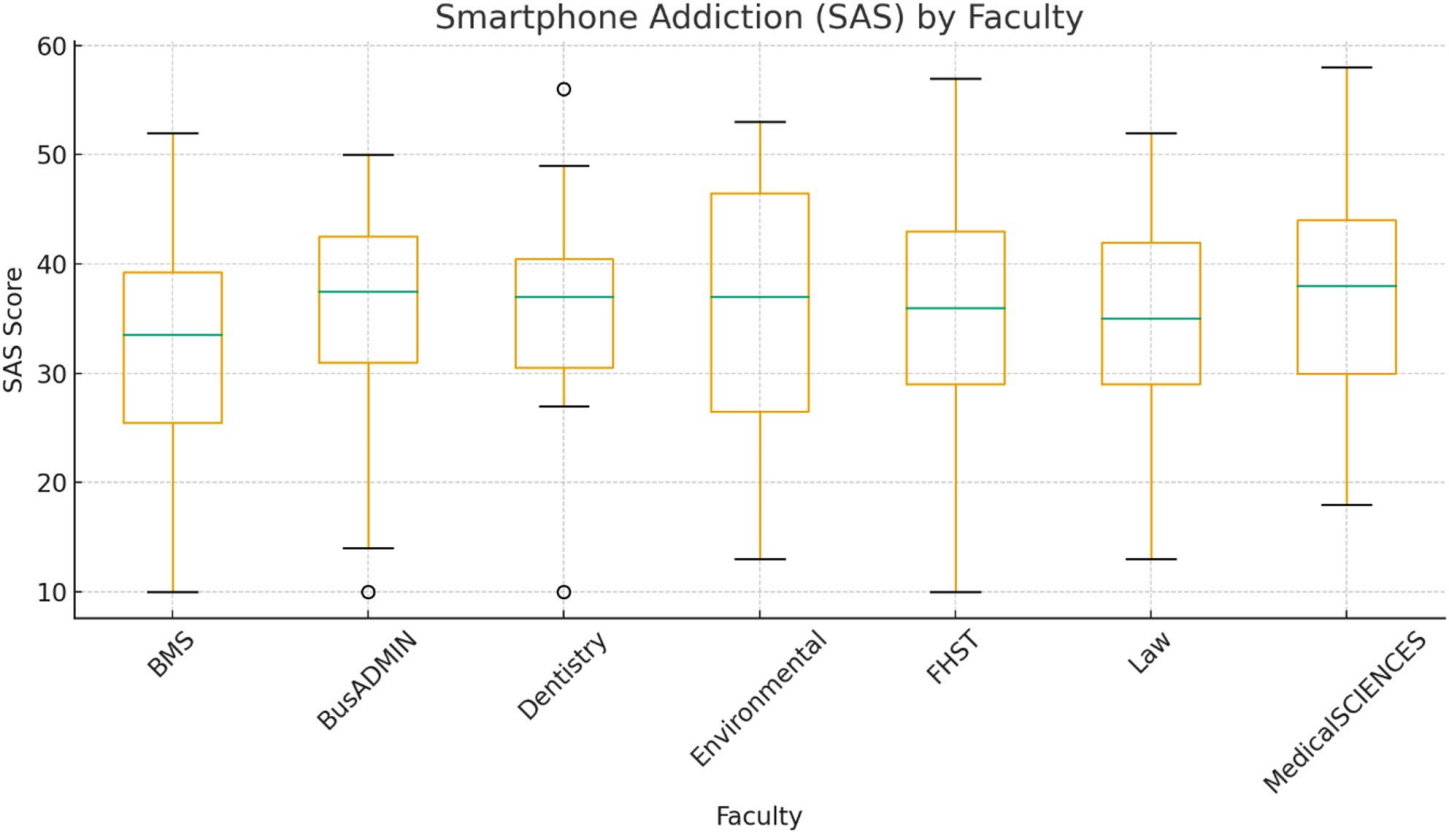
Box plot of smartphone addiction scores by faculty level. Notes: This plot shows clear variations in smartphone addiction across faculties. Business administration, health sciences and technology and environmental studies exhibit the highest distributions

**Fig. 5:**
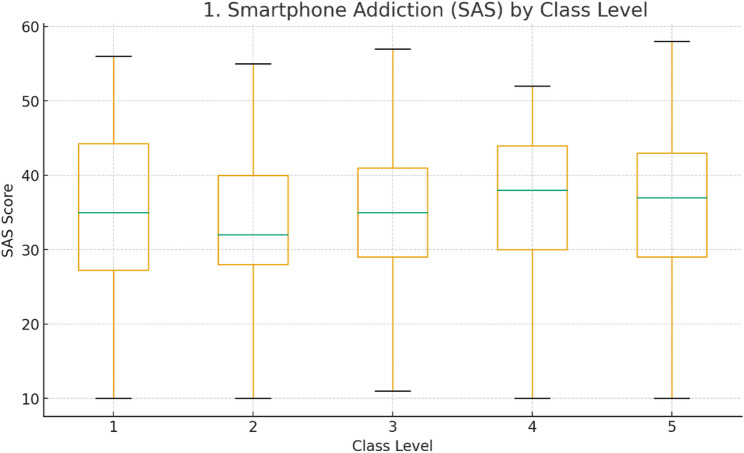
Box plot of smartphone addiction scores by academic level (Years 1–5), illustrating differences across class levels

**Fig. 6:**
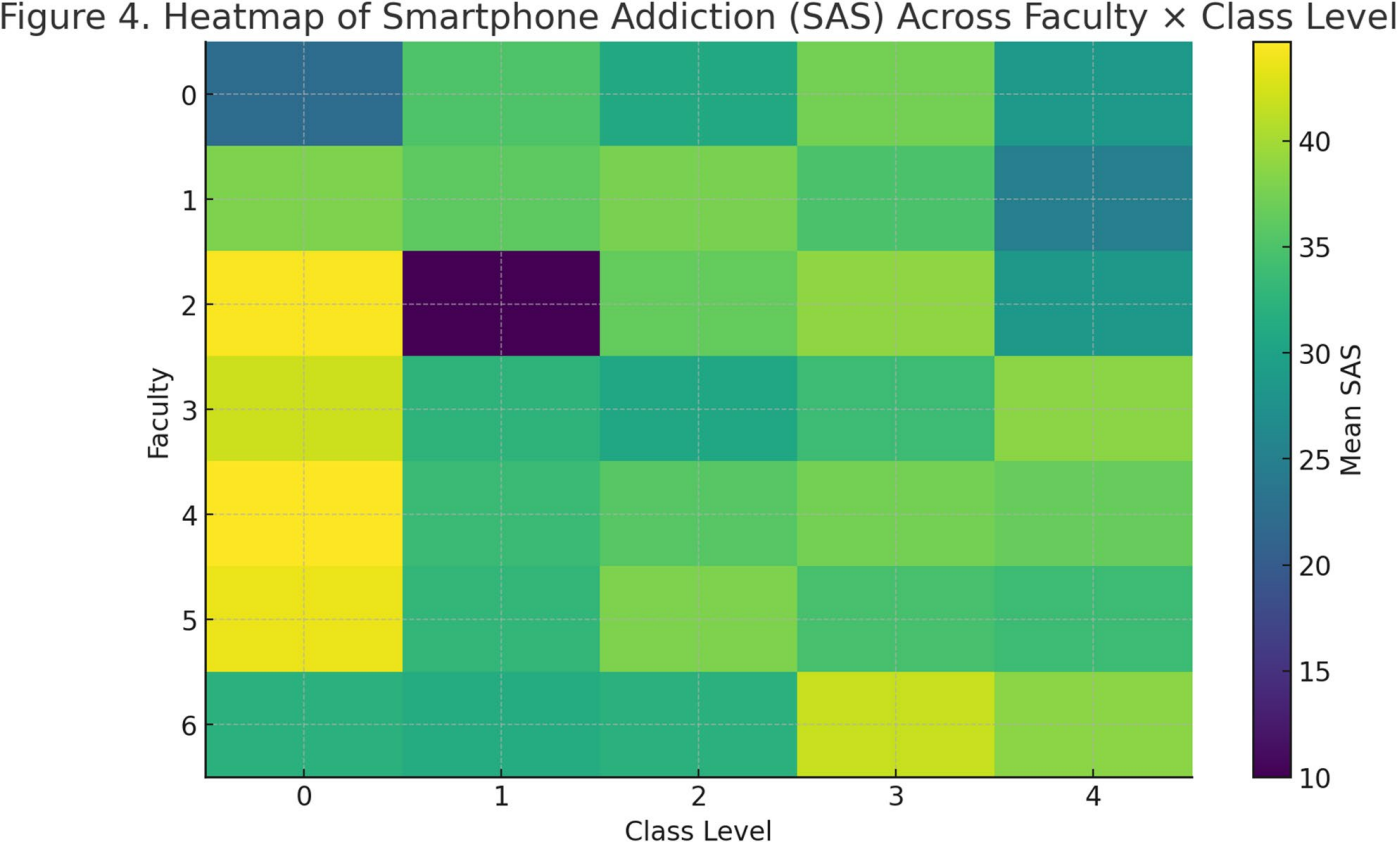
Heatmap of smartphone addiction scores across faculty and academic level, highlighting patterns and peak values. Notes: Clear peaks in Year 4 across almost all faculties. Notes: Clear peaks in Year 4 across almost all faculties

**Fig. 7:**
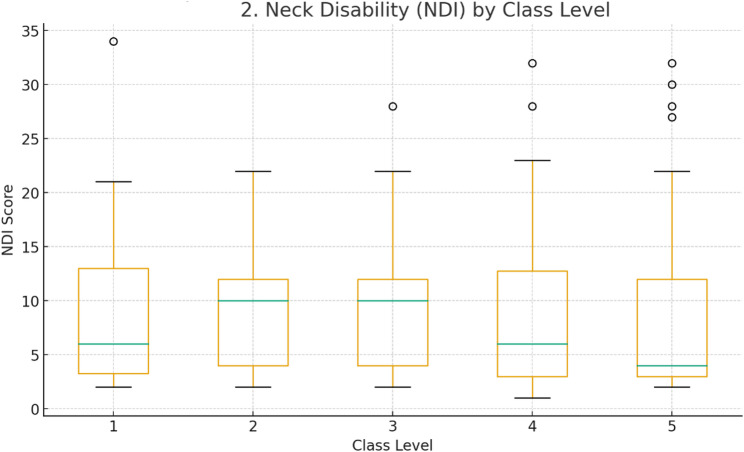
Box plot of neck-related functional disability scores by academic level.Notes: Neck-related functional disability increases across higher years, with more severe outliers in Years 4 and 5

**Fig. 8:**
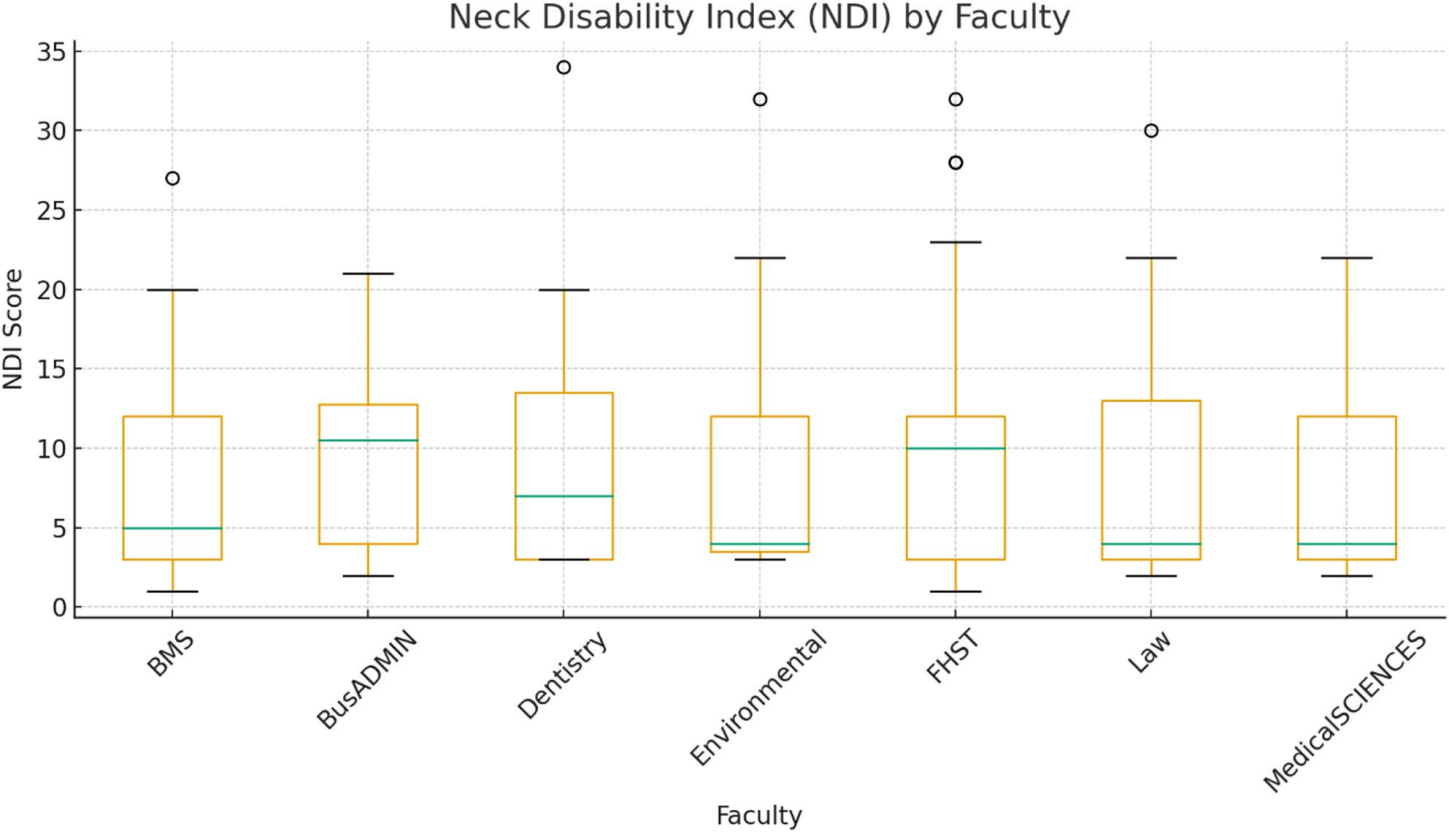
Box plot of neck-related functional disability scores by faculty. Notes: Neck-related functional disability varies significantly by faculty, with higher neck disability appearing in health sciences and technology, business administration, environmental studies, and law

**Fig. 9:**
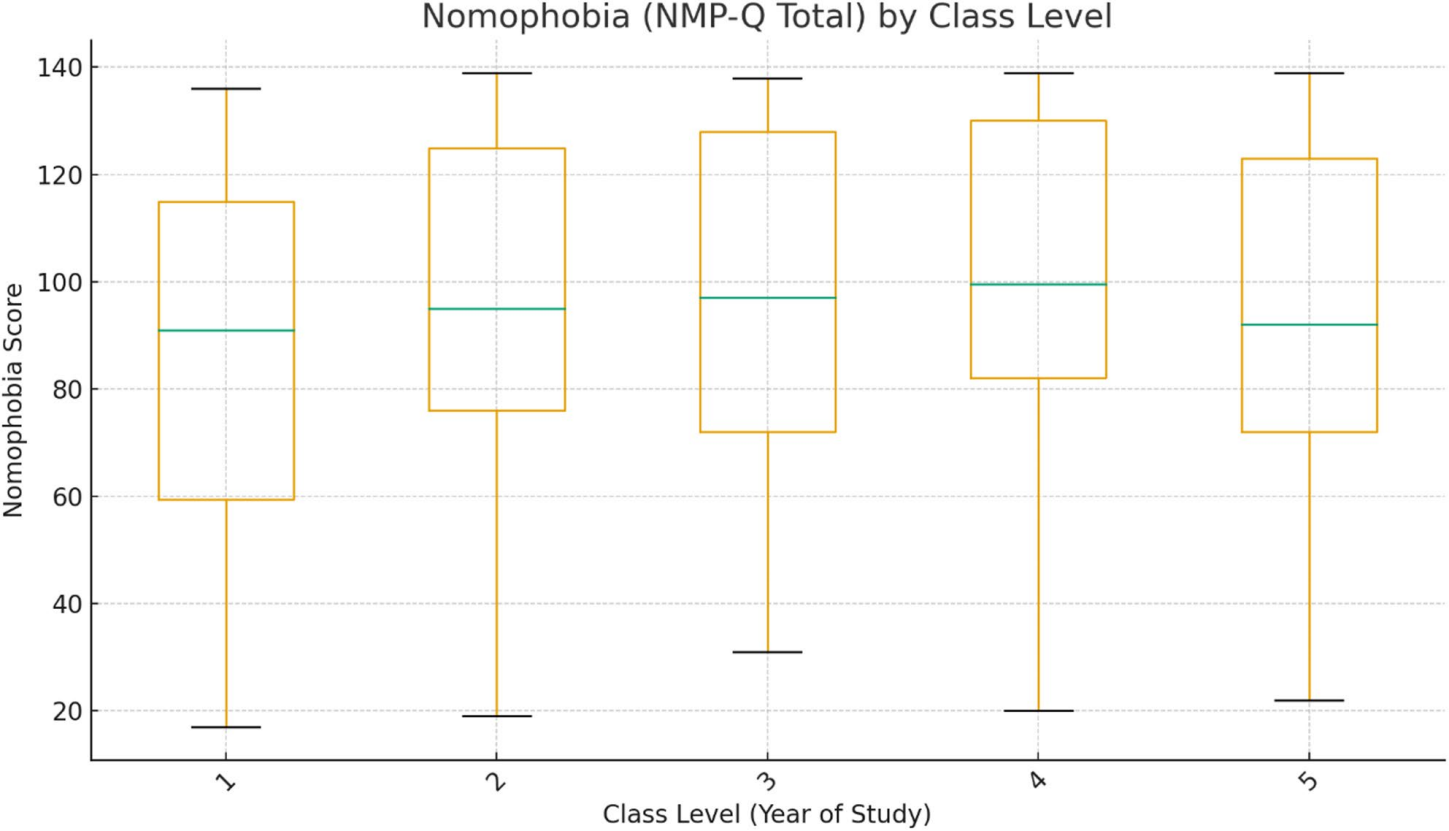
Box plot of nomophobia scores by academic level. Notes: Nomophobia scores increase progressively across academic levels, rising from Year 1 and peaking in Year 4 before showing a slight decline in Year 5. The distribution remains wide across all years, indicating consistently elevated nomophobia among undergraduate students

**Fig. 10:**
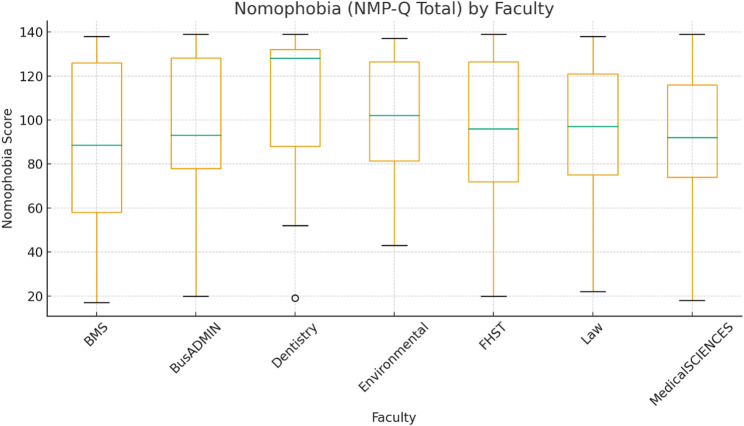
Box plot of nomophobia scores by Faculty. Notes: Nomophobia scores are notably higher in business administration, dentistry, health sciences and technology, and environmental studies

Clear and consistent patterns emerged when smartphone addiction, nomophobia, posture, and NFD were analysed by faculty and academic level (Figs. 4, 5, 6, 7, 8, 9 and 10):

### Academic-level trends

Academic-level trends showed that Years 4 and 5 consistently demonstrated the highest smartphone use hours, smartphone addiction, nomophobia, neck flexion angles, and neck-related functional disability. In contrast, Years 1–2 exhibited the lowest values across all behavioural and musculoskeletal variables.

*.*

#### Faculty-level trends

**Fig. 11:**
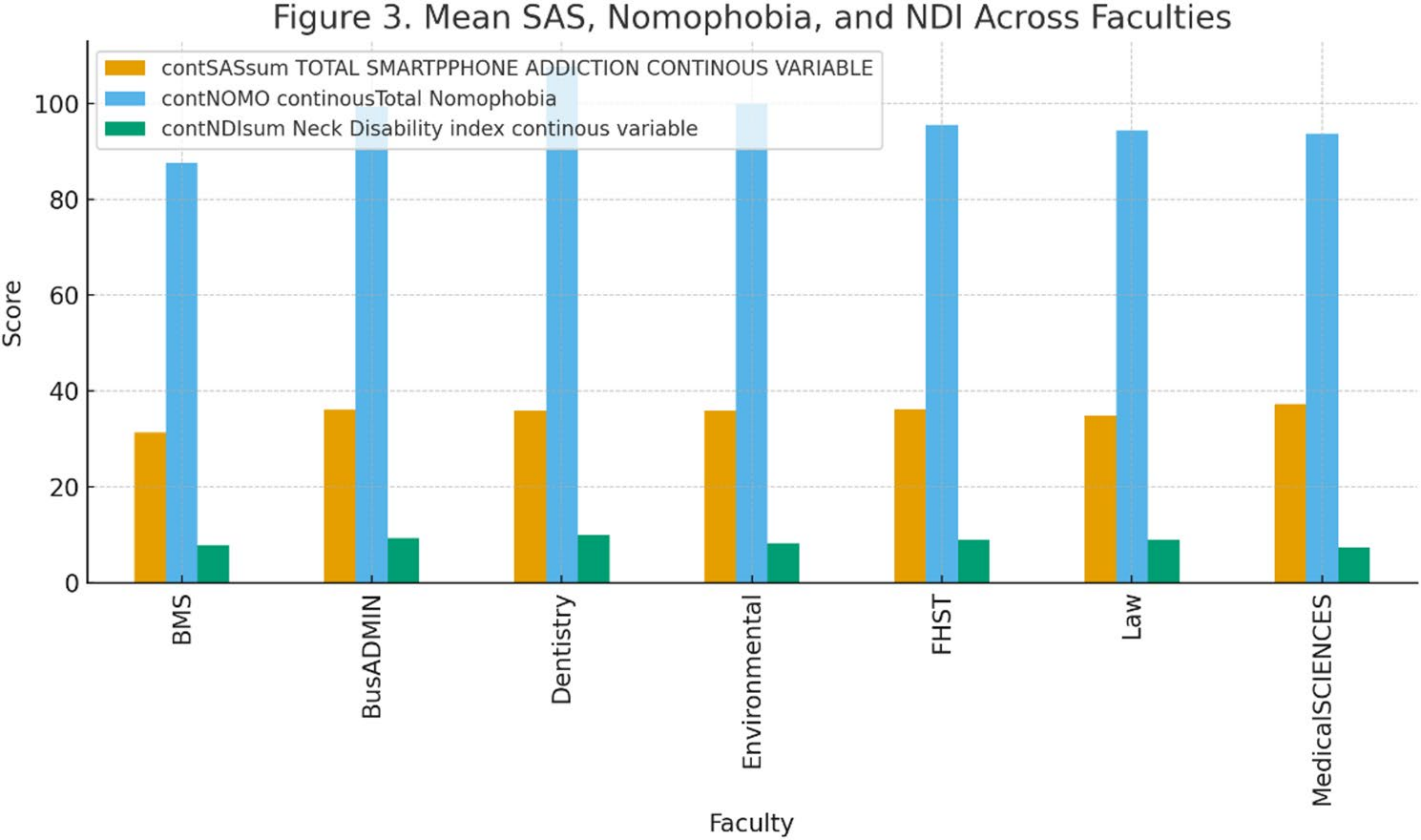
Mean smartphone addiction (contSASsum), Nomophobia (contNOMO), Neck-related functional Disability (contNDIsum) across faculties. Notes: Business Administration, Health Sciences and Technology, and Environmental Studies show the highest burden

Faculties with the highest cumulative digital and ergonomic burden included: Business Administration, Health Sciences and Technology, Environmental Studies, Dentistry and Medical Sciences. Figure 11 illustrates the mean levels of smartphone addiction, nomophobia, and NFD across different faculties, highlighting the variations in digital dependence and musculoskeletal symptoms.

### Cumulative exposure pattern

Across both faculty and academic-level analyses, findings strongly indicated a cumulative exposure trajectory: Higher academic levels were associated with higher smartphone dependence and poorer ergonomic behaviours, accompanied by increased NFD.

### Normality and transformation of continuous variables

Assessment of distributional properties showed that the NFD total score was substantially right-skewed, as indicated by the shapiro-wilk test (W = 0.856, *p* < 0.001), skewness (1.10), and kurtosis (1.09). To improve normality and stabilise variance, the NFD score was log-transformed to produce log NFD. The transformed variable demonstrated markedly improved distributional characteristics, with skewness near zero (0.01), reduced kurtosis (–1.23), and Q-Q plots that aligned more closely with the theoretical normal distribution. Accordingly, log NFD score was used as the dependent variable in all subsequent regression analyses.

Smartphone addiction and nomophobia scores demonstrated only mild departures from normality (skewness < 0.5; kurtosis < 1), despite statistically significant shapiro–wilk results due to the large sample size (Smartphone addiction: W = 0.986, *p* = 0.001; Nomophobia: W = 0.942, *p* < 0.001). Given their acceptable symmetry and theoretical interpretability, both variables were retained in their original scales for correlation and regression modelling. A summary of the normality tests for all continuous variables is presented in Table 11.

**Table 11:** Normality tests (shapiro–wilk, skewness and kurtosis) for continuous variables

Variable	*n*	Shapiro-wilk W	Shapiro-wilk *p*	Skewness	Kurtosis
NFD	399	0.856	< 0.0001	1.10	1.09
Log NFD	399	0.913	< 0.0001	0.01	−1.23
Smartphone addiction	399	0.986	0.00064	−0.32	−0.15
Nomophobia	399	0.942	< 0.0001	−0.44	−0.60

*NFD* Neck-related functional disability, log NFD - Neck-related functional disability (log transformed); Skewness and kurtosis are based on Fisher’s definition; *0 = perfectly normal*

### Objective 2 - associations between smartphone addiction, nomophobia, and neck-related functional disability

#### Pearson correlation analysis

The correlation analysis showed several statistically significant associations between digital dependence, posture-related behaviour, and NFD (Table 12). Smartphone addiction demonstrated a strong positive correlation with nomophobia (*r* = 0.556, *p* < 0.001), indicating substantial overlap between problematic smartphone use and anxiety related to being without the device. Smartphone addiction was also moderately correlated with daily smartphone use (*r* = 0.322, *p* < 0.001) and neck flexion angle (*r* = 0.326, *p* < 0.001), suggesting that higher addiction scores were associated with greater exposure time and more forward-flexed neck posture. Its association with NFD was smaller but statistically significant (*r* = 0.249, *p* < 0.001).

Nomophobia showed significant positive correlations with daily smartphone use (*r* = 0.251, *p* < 0.001) and neck flexion angle (*r* = 0.239, *p* < 0.001), but its correlation with NFD was small and not statistically significant (*r* = 0.087, *p* = 0.084) (Table 12). Neck-related functional Disability displayed its strongest correlation with neck flexion angle (*r* = 0.155, *p* = 0.002), supporting the role of posture as an ergonomic contributor to neck-related symptoms, while its association with hours of use was weak (*r* = 0.066, *p* = 0.186).

Hours of smartphone use per day correlated significantly with both smartphone addiction and nomophobia, but only weakly with NFD. Age and academic year showed small but significant associations with neck angle and with each other, but minimal relationships with smartphone addiction, nomophobia, or NFD. Overall, these findings suggest that smartphone addiction and forward-flexed posture are more consistently associated with neck-related symptoms than either nomophobia or duration of use alone.

**Table 12:** Pearson correlation coefficients among key study variables (*N* = 399)

Variable	SAS	Nomophobia	NFD	Hours/day	Neck angle	Age	Academic year
Smarttpone addiction	1.000	0.556***	0.249***	0.322***	0.326***	–0.019	0.097
Nomophobia	0.556***	1.000	0.087	0.251***	0.239***	–0.053	0.017
NFD	0.249***	0.087	1.000	0.066	0.155**	0.007	–0.069
Hours/day	0.322***	0.251***	0.066	1.000	0.118*	–0.076	0.008
Neck angle	0.326***	0.239***	0.155**	0.118*	1.000	0.139**	0.114*
Age	–0.019	–0.053	0.007	–0.076	0.139**	1.000	0.128*
Academic year	0.097	0.017	–0.069	0.008	0.114*	0.128*	1.000

*NFD* Neck-related functional Disability, Values are Pearson’s r. with *p* < 0.05*; ** *p* < 0.01; *** *p* < 0.001

#### Sensitivity analysis using spearman correlation

To account for the ordinal nature of some variables and potential deviations from normality, Spearman’s rank correlation coefficients were computed as a sensitivity analysis (Table 13). The Spearman results were broadly consistent with the Pearson correlation findings. Smartphone addiction remained significantly associated with nomophobia (ρ = 0.500, *p* < 0.001), neck-related functional disability (ρ = 0.283, *p* < 0.001), daily smartphone use (ρ = 0.333, *p* < 0.001), and neck flexion angle (ρ = 0.317, *p* < 0.001).

Nomophobia also demonstrated a weak but statistically significant association with neck-related functional disability (ρ = 0.104, *p* = 0.038). Neck flexion angle remained significantly associated with neck-related functional disability (ρ = 0.165, *p* = 0.001). Overall, the consistency of findings across both Pearson and Spearman analyses supports the robustness of the observed associations and indicates that smartphone addiction and forward-flexed posture are reliably associated with neck-related functional disability.

**Table 13:** Spearman rank correlation matrix among study variables (*N* = 399)

Variable	SAS	Nomophobia	NFD	Hours/day	Neck angle	Age	Academic level
Smartphone addiction	1.000	0.500***	0.283***	0.333***	0.317***	-0.014	0.073
Nomophobia	0.500***	1.000	0.104*	0.258***	0.238***	-0.088	-0.021
NFD	0.283***	0.104*	1.000	0.055	0.165***	0.017	-0.102*
Hours/day	0.333***	0.258***	0.055	1.000	0.128*	-0.135**	0.015
Neck angle	0.317***	0.238***	0.165***	0.128*	1.000	0.062	0.097
Age	-0.014	-0.088	0.017	-0.135**	0.062	1.000	0.124*
Academic year	0.073	-0.021	-0.102*	0.015	0.097	0.124*	1.000

*SAS* Smartphone addiction, *NFD* Neck-related Functional Disability; Values are spearman’s rho with *p* < 0.05*; ** *p* < 0.01; *** *p* < 0.001

### Objective 3 – multivariable associations between smartphone addiction, nomophobia and neck-related functional disability

To examine the extent to which smartphone addiction and nomophobia were associated with NFD, a series of multiple linear regression models was fitted with log-transformed NFD scores as the dependent variable. The initial full model included smartphone addiction, nomophobia, hours of smartphone use per day, break frequency, neck flexion angle, age, academic level, and sex.

#### Full multivariable model

In the full model (Table 14), smartphone addiction, neck flexion angle, and academic year were significantly associated with log NFD. Higher smartphone addiction scores and greater neck flexion angles were associated with higher NFD, whereas higher academic level was associated with slightly lower NFD after adjustment for other variables. Nomophobia, hours of smartphone use per day, break frequency, age, and sex were not significantly associated with NFD in this model.

**Table 14:** Full multiple linear regression model for log-transformed neck-related functional disability (log NFD) (*N* = 399)

Predictor	b	SE	t	*p*-value	95% CI
Intercept	0.374	0.292	1.28	0.201	–0.199 to 0.947
Smartphone addiction	0.0216	0.0035	6.18	< 0.001	0.015 to 0.028
Nomophobia	0.0002	0.0002	1.06	0.292	–0.0002 to 0.0006
Hours of smartphone use (per day)	0.0026	0.0079	0.33	0.738	–0.014 to 0.019
Break frequency	–0.0143	0.0192	–0.74	0.458	–0.052 to 0.024
Neck flexion angle (degrees)	0.0038	0.0016	2.31	0.022	0.001 to 0.007
Age (years)	–0.0023	0.0098	–0.23	0.816	–0.022 to 0.018
Academic level (1–5)	–0.0423	0.0148	–2.85	0.005	–0.072 to − 0.013
Sex (male vs. female)	0.0062	0.0392	0.16	0.875	–0.070 to 0.083

Model fit: R² = 0.125; adjusted R² = 0.107; AIC = 876.39.Model fit: R² = 0.125; adjusted R² = 0.107; AIC = 876.39

Outcome variable: log-transformed NDI total score

#### Backward elimination and stepwise models

A backward elimination procedure was used to remove non-significant variables from the full model. In the final backward model (Table 15), smartphone addiction, neck flexion angle, and academic level were retained as independent correlates of log NFD. Nomophobia, hours of smartphone use per day, break frequency, age, and sex were excluded due to lack of statistical contribution. A bidirectional stepwise procedure converged on the same three-predictor solution as the backward model, confirming the stability of this reduced set of correlates.

**Table 15:** Backward/stepwise final model for log-transformed neck-related functional disability (log NFD) (*N* = 399)

Predictor	b	SE	t	*p*-value	95% CI
Intercept	0.516	0.244	2.11	0.035	0.037 to 0.995
Smartphone addiction	0.0220	0.0033	6.63	< 0.001	0.015 to 0.029
Neck flexion angle (degrees)	0.0039	0.0017	2.36	0.019	0.001 to 0.007
Academic year (1–5)	–0.0411	0.0133	–3.09	0.002	–0.067 to − 0.015

Model fit: AIC = 870.49.: Outcome variable: log-transformed NDI total score

#### Forward selection model

In the forward selection strategy, starting from an intercept-only model, smartphone addiction was the only variable that met the entry criterion and improved model fit (Table 16). No other variable (including nomophobia, neck angle, hours of smartphone use per day, age, academic year, break frequency, or sex) further improved the model once smartphone addiction was included.

In the final model (Table 16), smartphone addiction remained a statistically significant correlate of log NFD (b = 0.0227, SE = 0.0036, t = 6.24, *p* < 0.001). This implies that a 10-point increase in smartphone addiction was associated with an approximately 25% higher expected NFD score (exp(0.227) ≈ 1.25). Nomophobia and demographic covariates did not materially improve model fit and were excluded for parsimony.

**Table 16:** Forward selection model for log-transformed neck-related functional disability (log NFD) (*N* = 399)

Predictor	b	SE	t	*p*-value
Intercept	1.093	0.135	8.10	< 0.001
Smartphone addiction	0.0227	0.0036	6.24	< 0.001

Model fit: AIC = 869.85. Outcome variable: log-transformed NFD total score

#### Comparison of model fit

To compare the competing models, Akaike information criterion (AIC) and Bayesian information criterion (BIC) was examined (Table 17). The forward model containing only smartphone addiction provided the lowest AIC and BIC, indicating the best balance between model fit and parsimony. The backward/stepwise model including smartphone addiction, neck angle, and academic level offered a slightly higher AIC/BIC, but added ergonomic and academic context to the association.

**Table 17:** Comparison of model fit indices for regression models predicting log neck-related functional disability

Model	Predictors included	AIC	BIC
Full model	Smartphone addiction, nomophobia, hours of device use, breaks, neck angle, age, academic year, sex	876.39	912.29
Backward model	Smartphone addiction, neck angle, academic year	870.49	886.45
Forward model	Smartphone addiction only	869.85	889.79
Stepwise (bidirectional) model	Smartphone addiction, neck angle, academic year	870.49	886.45

*AIC* Akaike information criterion, *BIC* Bayesian information criterion. Across all modelling strategies, smartphone addiction consistently emerged as the strongest independent correlate of NFD.

In every model where it was included, higher smartphone addiction scores were associated with higher log NFD values. Nomophobia did not show an independent association with NFD after accounting for smartphone addiction and other covariates.

Neck flexion angle showed a small but statistically significant positive association with NFD in the full and backward/stepwise models, suggesting that more forward-flexed posture is related to greater neck-related functional limitations. Academic level displayed a small inverse association with NFD in adjusted models, indicating slightly lower disability scores in higher academic years once smartphone addiction and posture were considered.

**Fig. 12:**
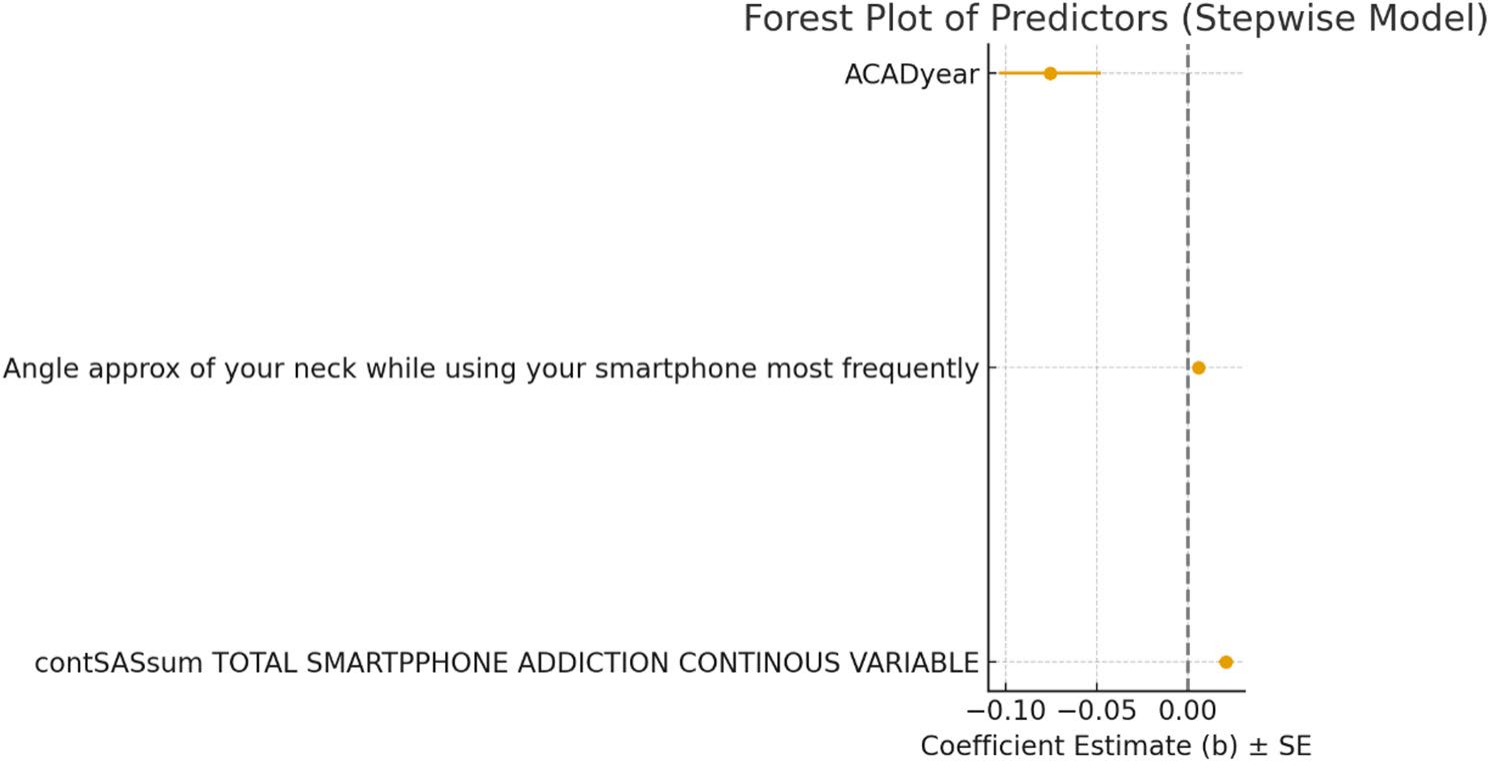
Forest Plot of Predictors (Stepwise Model)

A forest plot of the stepwise model (Fig. 12) illustrates these associations visually, with smartphone addiction showing the largest positive coefficient and narrow confidence intervals, neck angle showing a smaller positive association, and academic level showing a negative association with confidence intervals that do not cross zero.

Overall, the regression analyses indicate that behavioural dependence on smartphones (addiction) is the principal correlate of NFD in this sample, with posture-related strain (neck flexion) and academic stage providing additional but more modest explanatory contributions. All interpretations are based on cross-sectional associations and do not imply causality.

#### Model diagnostics

**Fig. 13:**
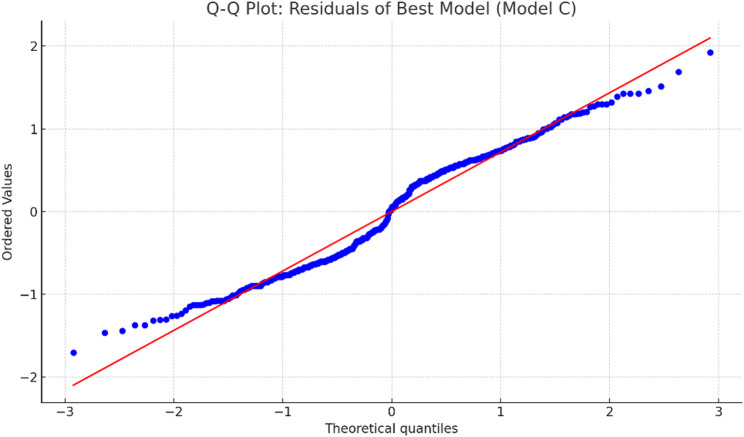
Q-Q Plot: Residuals of Best Model (Model C)

**Fig. 14:**
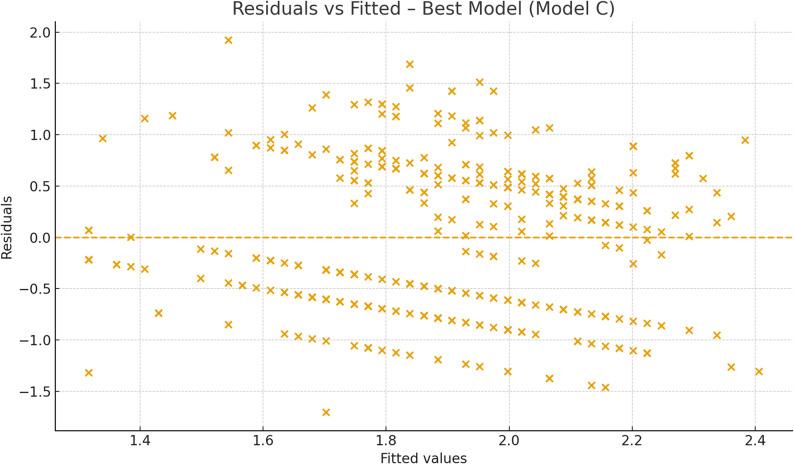
Residuals vs. Fitted – Best Model

Residual diagnostics supported the adequacy of the final models (Figs. 13 and 14). Residuals were symmetrically distributed around zero (median = 0.06; SD = 0.72), with no evidence of heteroscedasticity or severe non-linearity. Residuals from the final model were approximately symmetrically distributed (median 0.06, IQR − 0.62 to 0.59; SD 0.72; Table 18). Q–Q plots showed only mild tail deviations, and residuals versus fitted values displayed no systematic pattern, supporting the assumptions of normality and homoscedasticity. No influential outliers were detected.

**Table 18:** Residual distribution for the final regression model (Model C: log NFD ~ Smartphone addiction; *N* = 399)

Statistic	Value
Minimum	–1.70
1st Quartile (Q1)	–0.62
Median	0.06
3rd Quartile (Q3)	0.59
Maximum	1.92
Mean	~ 0.00
Standard Deviation	0.72

Residuals from the final model were approximately centered around zero with a symmetric spread, indicating no systematic over- or under-prediction. Q–Q plots showed only mild deviation from normality in the tails

Overall, the linear regression analyses highlight behavioural and ergonomic correlates of NFD across the full range of scores. The regression models explained a modest proportion of variance in NFD (R² ≈ 0.09–0.13), indicating that the behavioural and ergonomic variables included in this study capture only part of the factors influencing neck disability in this population. This level of explained variance is consistent with findings from cross-sectional behavioural and musculoskeletal research, where outcomes are typically influenced by a complex interplay of physical, psychological, and environmental determinants. Despite the modest explanatory power, smartphone addiction remained a consistent and statistically significant predictor across all modelling approaches, supporting the robustness of its association with neck-related functional outcomes.

### Binary logistic regression analysis

To provide clinically interpretable estimates of risk, a binary logistic regression analysis was conducted using moderate-to-severe neck disability (NDI ≥ 15) as the outcome variable. As shown in Table 19, smartphone addiction was a significant predictor of moderate-to-severe neck disability (OR = 1.04, 95% CI: 1.02–1.07, *p* < 0.001), indicating that higher smartphone addiction scores were associated with increased odds of clinically relevant disability. Neck flexion angle also demonstrated a significant association (OR = 1.03, 95% CI: 1.01–1.05, *p* = 0.018), suggesting that greater forward-flexed posture increases the likelihood of neck disability. In contrast, nomophobia, hours of smartphone use, academic level, age, and sex were not independently associated with moderate-to-severe disability. The model demonstrated acceptable fit (Nagelkerke R² ≈ 0.11), indicating that behavioural and ergonomic factors explain a modest proportion of the variability in clinically relevant neck disability. These findings complement the linear regression results by demonstrating that smartphone addiction and neck posture are not only associated with higher disability scores but also increase the likelihood of clinically meaningful impairment.

**Table 19:** Binary logistic regression analysis predicting moderate-to-severe neck disability (NDI ≥ 15) (*N* = 399)

Predictor	OR	95% CI	*p*-value
Smartphone addiction	1.04	1.02–1.07	< 0.001**
Nomophobia	1.00	0.99–1.01	0.311
Neck flexion angle	1.03	1.01–1.05	0.018*
Hours of smartphone use per day	1.02	0.95–1.10	0.521
Academic level	0.93	0.82–1.05	0.224
Age	1.01	0.89–1.14	0.872
Sex (male vs. female)	1.08	0.68–1.72	0.742

Model fit: Nagelkerke R² ≈ 0.11; Hosmer–Lemeshow *p* > 0.05 (good fit)

To further examine the impact of smartphone-use heterogeneity, hours of device use (per day) was modelled as a categorical variable, as presented in Table 20. Compared with moderate users, students in the very high-use category showed increased odds of moderate-to-severe neck disability; however, this association did not reach statistical significance (OR = 1.68, *p* = 0.096). These findings suggest a trend toward increased risk at higher exposure levels, although behavioural dependence (smartphone addiction) remained the dominant predictor.

**Table 20:** Logistic regression including smartphone use categories

Predictor	OR	95% CI	*p*-value
Smartphone addiction	1.04	1.02–1.07	< 0.001**
Neck flexion angle	1.03	1.01–1.05	0.018*
High use (7–9 h)	1.32	0.78–2.21	0.292
Very high use (≥ 10 h)	1.68	0.91–3.10	0.096
Moderate use (ref)	—	—	—

### Summary of key findings

Across all analyses, smartphone addiction emerged as the strongest and most consistent correlate of NFD. Forward-flexed posture also contributed significantly, although its effect was more modest in comparison. In contrast, nomophobia did not show an independent association with NFD once smartphone addiction was accounted for in the models. Academic level demonstrated a small inverse association, suggesting the possibility of adaptation or behavioural modification in later years of study.

Overall, these findings highlight the concurrent influence of behavioural dependence and posture-related mechanical load in explaining NFD among university students.

## Discussion

### Overview of key findings

This study examined patterns of smartphone use and posture-related behaviours among undergraduate students, and explored their associations with NFD. Three key findings emerged. First, university students demonstrated high daily smartphone use, frequent forward-flexed neck postures, and generally mild but notable levels of NFD. Second, smartphone addiction showed strong associations with nomophobia, smartphone use intensity, and neck flexion angle, and a moderate association with NFD. Third, regression modelling identified smartphone addiction as the strongest independent correlate of NFD, whereas nomophobia did not remain significant once addiction was accounted for. Neck flexion angle also demonstrated a small but meaningful association with NFD. Together, these findings highlight the combined behavioural and ergonomic risk profile associated with smartphone use in this population.

These findings reinforce global concerns that university students represent a high-risk group for digital-behavioural dependence and cervical musculoskeletal strain, as their academic, social, and recreational routines increasingly converge around smartphone-based tasks [1, 2]. The concurrence of heavy device use and posture-related stress observed in this study therefore reflects not only individual behaviour but also broader shifts in digital learning environments and youth communication patterns.

### Interpretation in relation to existing literature

#### Smartphone use patterns and posture

The high daily smartphone use documented in this study is consistent with global trends showing increasing reliance on mobile technologies among university students [37, 38]. The predominance of forward-flexed neck postures (30°–60°) similarly aligns with findings from earlier ergonomic investigations, which report that smartphone users commonly maintain sustained flexed positions that elevate mechanical loading on the cervical spine [39, 40]. Flexion angles exceeding 30° markedly increase compressive forces on the cervical vertebrae and surrounding soft tissues, as demonstrated in biomechanical modelling studies [5], predisposing individuals to discomfort and fatigue. This aligns with previous research indicating that high volumes of smartphone use in young adults are associated with musculoskeletal symptoms, particularly neck pain and disability [9, 10]. Several recent studies suggest that young adults often underestimate the degree of neck flexion adopted during smartphone use, which may partially explain the high prevalence of text neck–related symptoms, even among individuals who perceive their posture as acceptable [22].

Environmental factors such as poorly designed lecture halls, inadequate seating, and prolonged screen-based academic tasks may further promote forward-flexed postures, contributing to cumulative cervical strain [6]. Consequently, the posture patterns observed in this study closely reflect established biomechanical risk pathways.

#### Smartphone addiction and nomophobia

The moderate-to-high levels of smartphone addiction and nomophobia observed in this sample mirror evidence from studies conducted in Asia, the Middle East, and Africa [25, 41, 42]. The strong relationship between smartphone addiction and nomophobia is well established, with both constructs reflecting closely overlapping behavioural tendencies, including compulsive checking, fear of disconnection, and difficulty disengaging from online activities. This alignment is further reinforced by studies documenting their frequent co-occurrence and highlighting how these shared behaviours contribute to the interconnected nature of the two constructs [43, 44].

Beyond these behavioural overlaps, emerging evidence suggests that heightened nomophobia may be driven not only by behavioural factors but also by psychological mechanisms such as anxiety sensitivity, low tolerance for uncertainty, and reliance on digital communication for emotional regulation [45, 46]. These mechanisms may interact with behavioural dependence, intensifying the reinforcing cycle between smartphone addiction and nomophobia, especially in academic environments where constant connectivity is seen as essential. Consequently, these overlapping features offer a more integrated explanation for their consistent co-occurrence observed in this study.

Although nomophobia was significantly correlated with smartphone addiction and behavioural use patterns, it did not independently predict NFD in multivariable models. This finding suggests that nomophobia may reflect psychological dependence on smartphone connectivity rather than a direct driver of musculoskeletal strain. The strong association between nomophobia and smartphone addiction observed in this study is consistent with previous literature demonstrating substantial conceptual overlap between these constructs, including compulsive checking behaviours, anxiety related to disconnection, and persistent engagement with digital environments. However, when behavioural intensity (smartphone addiction) was accounted for, nomophobia did not contribute additional explanatory value to NFD outcomes.

This pattern indicates that the physical consequences of smartphone use may be more closely linked to behavioural manifestations of dependence such as prolonged usage duration and sustained poor posture rather than the psychological experience of disconnection itself. In this context, nomophobia may operate indirectly, influencing musculoskeletal risk through its association with addictive use behaviours rather than exerting a direct effect. This distinction highlights the importance of differentiating between psychological dependence and behaviourally mediated physical risk in digital health research. The observed pattern is consistent with a potential mediation framework in which nomophobia influences smartphone addiction, which in turn affects usage behaviour and posture, ultimately contributing to NFD. Although formal mediation analysis was beyond the scope of this study, this pathway warrants further investigation in longitudinal designs.

### Associations with neck-related functional disability

Neck-related functional disability in this study was generally mild but increased with higher smartphone addiction scores and advancing academic levels. This trend is consistent with previous research indicating that musculoskeletal symptoms are more prevalent among individuals who engage in heavy or problematic smartphone use [47, 48], with similar positive associations reported in studies conducted in Saudi arabia, India, and South Africa [49, 50].

Within our sample, the neck flexion angle emerged as the strongest ergonomic factor associated with NFD, aligning with existing literature highlighting cervical posture as a mediator in the relationship between smartphone exposure and musculoskeletal strain [13, 51]. Regression modeling identified smartphone addiction as the most significant independent predictor of NFD, whereas nomophobia did not remain significant once addiction was considered. The binary logistic regression analysis further strengthens the findings by demonstrating that smartphone addiction is not only associated with higher neck disability scores but also predicts the likelihood of clinically meaningful disability. This reinforces the role of behavioural dependence as a key determinant of musculoskeletal risk, beyond simple exposure duration. Consistent with the linear models, nomophobia did not independently predict disability, suggesting that its effects may operate indirectly through addictive behaviour patterns. The neck flexion angle also showed a small yet meaningful association with NFD, underscoring the importance of ergonomic factors. This pattern reflects emerging evidence that even mild NFD in young adults may predict later complications if high-risk behaviours persist [36], and that cumulative exposure, rather than single-session duration, is a more reliable predictor of neck disability [52]. The regression models demonstrated modest explanatory power, consistent with the multifactorial nature of musculoskeletal conditions, which are influenced by a complex interplay of behavioural, ergonomic, psychological, and environmental factors.

Importantly, despite the relatively low R² values, smartphone addiction emerged as a consistent and statistically robust correlate across all modelling approaches, including linear and logistic regression. This suggests that behavioural dependence on smartphone use may represent a key modifiable risk factor, even within a broader network of unmeasured influences such as physical activity, stress, sleep quality, and workstation ergonomics. These findings align with prior research indicating that behavioural variables often account for modest proportions of variance in health outcomes, yet remain clinically meaningful due to their modifiability and population-level impact.

The modest variance explained by the models should therefore be interpreted not as a limitation of the analytical approach, but as an indication of the complex and multidimensional nature of neck-related musculoskeletal outcomes in young adults.

### Comparison of findings with African and international studies

The findings align with emerging African evidence documenting a high prevalence of neck-related musculoskeletal symptoms and problematic smartphone use among university students [18–20, 50]. The progressive increase in addiction, nomophobia, and NFD across academic levels parallels observations in Ethiopia, South Korea, Italy, and India, where upper-level students report more entrenched digital behaviours and higher musculoskeletal symptom burden [53–56]. These parallels suggest that the behavioural and ergonomic patterns in this Nigerian cohort reflect a broader global phenomenon among contemporary students. Similar trends have been noted in China, Malaysia, and Turkey, where later-year students demonstrate heavier reliance on smartphones for academic content delivery, online assessments, and professional networking [15, 57–59], This indicates that digital dependence is likely reinforced not only by personal habits but also by structural academic requirements, which may explain the consistent escalation across year groups observed in this study.

### Pathways connecting behavioural dependence and text neck

#### Behavioural mechanisms

Smartphone addiction may influence neck-related symptoms through behavioural pathways that increase both the duration and intensity of device engagement [9–11, 46–48]. Addicted users engage in frequent, prolonged sessions characterised by compulsive scrolling, repeated checking, and reduced attentional control over posture [60–62], limiting opportunities for micro-breaks that allow postural adjustment and cervical muscle recovery [48]. Over time, this leads to prolonged exposure to ergonomically unfavourable positions, increasing the risk of discomfort or disability [5, 13, 23].

Additionally, behavioural studies indicate that individuals with high smartphone addiction often show diminished interoceptive awareness, reflecting a reduced ability to detect bodily discomfort or fatigue [63]. This may delay postural correction and accelerate musculoskeletal strain [64], further strengthening the behavioural pathway linking addiction to neck–related musculoskeletal symptoms.

#### Ergonomic mechanisms

Ergonomic factors play a central role in the development of text-neck symptoms. Forward-flexed neck postures increase mechanical load on the cervical spine, with biomechanical modelling indicating that at 30° of neck flexion the effective head weight increases to approximately 18 kg, and at 60° it approaches 27 kg [5]. This increased load places continuous tension on cervical musculature, ligamentous structures, and intervertebral discs, and prolonged maintenance of such positions is associated with muscle fatigue, impaired proprioception, and accumulated strain, contributing to discomfort and functional limitation [13, 23, 47].

Recent electromyographic studies indicate that forward-flexed posture increases upper trapezius and levator scapulae activation even during passive smartphone viewing, confirming persistent submaximal muscle loading [47, 65]. Over time, this sustained activation may contribute to pain, altered cervical alignment, and reduced neuromuscular control [66, 67].

### Combined behavioural–ergonomic risk model

The results of this study support an integrated behavioural–ergonomic framework for understanding NFD. Smartphone addiction appears to heighten ergonomic risk by increasing total exposure time and reducing posture awareness, thereby amplifying the duration spent in forward-flexed positions [60]. Neck flexion angle, in turn, represents the biomechanical pathway through which behavioural dependence manifests as physical strain [5, 13]. Although nomophobia demonstrated strong correlations with addiction, it did not independently predict NFD once addiction was accounted for, suggesting that its influence may operate indirectly through behavioural reinforcement rather than direct musculoskeletal mechanisms.

Together, these pathways highlight the interplay between digital behaviours and physical posture, offering a more comprehensive understanding of how problematic smartphone use may contribute to the development of neck-related symptoms in university students. This integrated perspective aligns with emerging digital-health models that conceptualize musculoskeletal disorders related to technology use as multifactorial syndromes involving behavioural, ergonomic, psychological, and environmental determinants [8, 38, 48]. By situating addiction and posture within a shared risk model, the present findings contribute to a growing body of work emphasizing the need for interdisciplinary prevention strategies.

### Implications for public health, universities and digital-wellness policy

#### Need for screening and early identification

The high prevalence of smartphone addiction and nomophobia identified in this study suggests that routine screening within university health services may be warranted. Validated instruments, such as the SAS-SV and NMP-Q, could help identify students at elevated risk for behavioural dependence and associated musculoskeletal symptoms. Early identification may enable timely referral for counselling, behavioural interventions, or ergonomic guidance.

#### Smartphone-use education

Educational programmes targeting safe and healthy patterns of smartphone use represent an important preventive strategy. University-wide campaigns could highlight the risks associated with prolonged device use, compulsive checking behaviour, and sustained forward-flexed posture. Digital-wellness interventions, such as workshops or awareness modules, have been shown to reduce problematic smartphone use and enhance ergonomic behaviours among students [68, 69]. Embedding such content into orientation programmes or general studies curricula may improve reach and effectiveness.

#### Posture correction and ergonomic training

Ergonomic education is essential, given the strong association between neck flexion angle and NFD observed in this study. Training students to maintain neck-neutral postures, elevate devices to eye level, and incorporate regular postural breaks may reduce cervical loading [5, 13, 65]. Integrating ergonomic modules into student health courses, physiotherapy outreach, or campus wellness initiatives could promote safer device-use habits. Practical demonstrations and infographic-based learning may further facilitate adoption.

#### Digital hygiene interventions

Digital hygiene practices, such as screen-time limits, app-usage tracking, scheduled “phone-free” study intervals, and mindfulness-based approaches, may mitigate compulsive smartphone use and encourage healthier engagement patterns [68, 69]. Universities could support these efforts by providing access to digital-wellness apps, structured study-skills coaching, or brief cognitive-behavioural strategies aimed at reducing problematic use.

#### Contextual implications for Nigerian university settings

Within Nigerian universities, the musculoskeletal burden associated with smartphone use may be amplified by contextual factors, including high digital demands across academic programmes and limited ergonomic infrastructure in libraries, lecture halls, and dormitories. Poorly designed seating, inadequate lighting, and overcrowded study spaces exacerbate forward-flexed posture and discomfort. Institutional responses should therefore include ergonomic improvements to study environments, incorporation of digital-wellness policies into student support systems, and collaboration between university health units, physiotherapy departments, and ICT units to deliver integrated interventions. Such measures could help mitigate the behavioural and ergonomic risks identified in this study and support healthier digital engagement among students.

### Strengths and limitations

#### Strengths

This study has several methodological strengths. First, it draws on a large and diverse sample of undergraduate students across all academic levels and faculties, enhancing internal representativeness within the institution. Second, the study employed validated and widely used instruments to assess smartphone addiction, nomophobia, and neck disability, strengthening measurement reliability. Third, the analytical strategy was rigorous: normality was assessed and appropriately corrected, and a combination of correlation analysis and multivariable regression including systematic model comparison was used to evaluate the relationships among behavioural and ergonomic variables. Together, these features enhance the robustness of the findings.

#### Limitations

Several limitations should be acknowledged when interpreting the results. The cross-sectional design prevents establishment of temporal ordering and causal inference. All primary variables, including smartphone use, posture, and musculoskeletal symptoms, were self-reported, introducing the potential for recall bias and social desirability bias. Convenience sampling also limits generalisability beyond the study population. The study-developed smartphone-use and posture items were designed to capture descriptive behavioural and ergonomic exposure variables rather than a single latent construct. Although these items were reviewed for clarity and pilot-tested before use, formal psychometric validation was not conducted. In addition, neck posture was assessed via self-reported visual estimation rather than objective biomechanical measurement, which may have introduced measurement imprecision. These constraints may have led to conservative or imprecise estimates of associations and should be considered in interpretation. Despite these limitations, the findings remain consistent with prior literature and provide useful insights into behavioural and ergonomic risk factors among university students.

### Future research directions

Future studies should adopt longitudinal designs to track smartphone-use behaviour, posture habits, and musculoskeletal outcomes over time, enabling stronger inferences about temporal associations. Intervention-based or experimental studies are needed to evaluate the effectiveness of ergonomic training, posture-correction strategies, digital hygiene programmes, and smartphone-addiction reduction interventions. Further research should also explore psychosocial moderators such as stress, coping strategies, academic workload, and sleep quality that may influence the relationship between digital dependence and musculoskeletal outcomes. Incorporating biomechanical tools (e.g. inclinometers or motion-tracking) could improve precision in posture assessment.

## Conclusion

Undergraduate students in this setting exhibited high levels of smartphone use, frequent forward-flexed posture during device interaction, and mild but notable NFD. Smartphone addiction emerged as the strongest behavioural correlate of NFD, whereas nomophobia demonstrated a secondary association that did not independently predict NFD in multivariable models. Neck flexion angle was identified as an important ergonomic correlate, indicating that both behavioural dependence and posture contribute meaningfully to neck symptoms. These findings should be interpreted as associative rather than causal and reflect behavioural and ergonomic correlates observed within a single university population. These findings highlight the need for integrated behavioural and ergonomic strategies, including digital-wellness education, addiction-mitigation approaches, and posture training, to promote healthier smartphone use and reduce musculoskeletal burden among university populations.

## Supplementary Information

Supplementary Material 1.

Supplementary Material 2.

Supplementary Material 3.

## Data Availability

The datasets used and/or analysed during the current study are available from the corresponding author on reasonable request ( [cynthia.john@unn.edu.ng](mailto: cynthia.john@unn.edu.ng) ).

## Abbreviations

SAS-SV: Smartphone Addiction Scale- Short version
NMP-Q: Nomophobia Questionnaire
NFD: Neck-related Functional Disability
NDI: Neck Disability Index
UNEC: University of Nigeria Enugu Campus
AIC: Akaike Information Criterion
BIC: Bayesian Information Criterion
