# Supplementary figures and images for: Smartphone addiction, nomophobia, and neck-related functional disability among undergraduate students in a Nigerian University: a cross-sectional study
Source: BMC Public Health. 2026 May 28;26:2153. doi: 10.1186/s12889-026-27976-z (PMC13371216; doi:10.1186/s12889-026-27976-z)

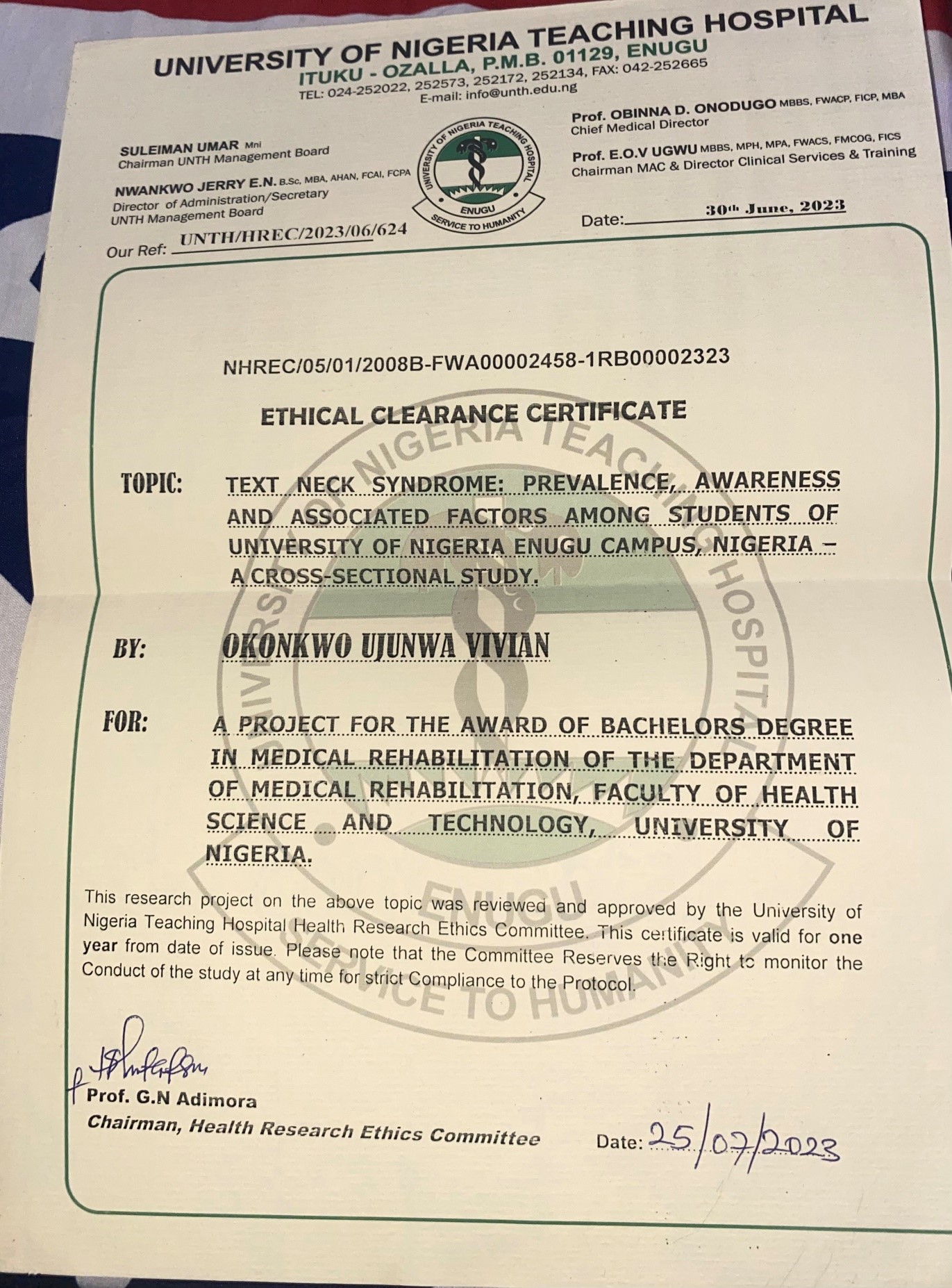

Supplement: Supplementary file 2 — Supplementary Material 2. [file 12889_2026_27976_MOESM2_ESM.jpg]
