# Supplementary material for: Smartphone addiction, nomophobia, and neck-related functional disability among undergraduate students in a Nigerian University: a cross-sectional study
Source: BMC Public Health. 2026 May 28;26:2153. doi: 10.1186/s12889-026-27976-z (PMC13371216; doi:10.1186/s12889-026-27976-z)

# Supplementary File 1: Study-Developed Questionnaire

## SECTION 1: SOCIO-DEMOGRAPHIC AND SMARTPHONE USE CHARACTERISTICS

## Section 1A: Socio-demographic Characteristics

1. Age (in years): ________

2. Sex: ☐ Male ☐ Female

3. Faculty/College: ____________________________

4. Department/Programme: _______________________

5. Academic year/level: ☐ Year 1 ☐ Year 2 ☐ Year 3 ☐ Year 4 ☐ Year 5

## Section 1B: Smartphone Use Characteristics

6. On average, how many hours per day do you use your smartphone? ________ hours/day

7. How often do you take breaks while using your smartphone?

☐ Never

☐ Rarely (less than once per session)

☐ Sometimes (about once per session)

☐ Often (more than once per session)

☐ Always (regular breaks every session)

## Section C: Neck Posture During Smartphone Use

8. Please select the image or description below that best represents your usual neck position when using your smartphone.

☐ 0° (Neutral): Head upright; phone held at or slightly below eye level

☐ 15° (Slight tilt): Small forward tilt; phone slightly below eye level

☐ 30° (Mild bend): Moderate forward tilt; phone held around chest level

☐ 45° (Moderate bend): Pronounced forward tilt; phone held around mid-abdomen or upper-waist level

☐ 60° (Pronounced bend): Severe forward tilt; phone held near lap or lower abdomen


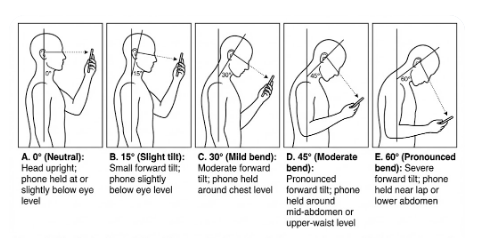

Supplement: Supplementary file 3 — Supplementary Material 3. [file 12889_2026_27976_MOESM3_ESM.docx]
